# Oscillations without cortex: Working memory modulates brainwaves in the endbrain of crows

**DOI:** 10.1016/j.pneurobio.2022.102372

**Published:** 2022-12

**Authors:** Lukas Alexander Hahn, Dmitry Balakhonov, Mikael Lundqvist, Andreas Nieder, Jonas Rose

**Affiliations:** a Neural Basis of Learning, Institute of Cognitive Neuroscience, Faculty of Psychology, Ruhr University Bochum, 44801 Bochum, Germany; b Department of Psychology, Department of Clinical Neuroscience, Karolinska Institute, Solna, Sweden; c Animal Physiology, Institute of Neurobiology, University of Tübingen, 72076 Tübingen, Germany

**Keywords:** AP, anterior-posterior axis, DVA, degrees of visual angle, LFP, local field potential, ML, medio-lateral axis, NCL, Nidopallium caudolaterale, PFC, prefrontal cortex, WM, working memory, Oscillations, Cognition, Crow, Gamma, Bursts, Evolution

## Abstract

Complex cognition requires coordinated neuronal activity at the network level. In mammals, this coordination results in distinct dynamics of local field potentials (LFP) central to many models of higher cognition. These models often implicitly assume a cortical organization. Higher associative regions of the brains of birds do not have cortical layering, yet single-cell correlates of higher cognition are very similar to those found in mammals. We recorded LFP in the avian equivalent of prefrontal cortex while crows performed a highly controlled and cognitively demanding working memory task. We found signatures in local field potentials, modulated by working memory. Frequencies of a narrow gamma and the beta band contained information about the location of target items and were modulated by working memory load. This indicates a critical involvement of these bands in ongoing cognitive processing. We also observed bursts in the beta and gamma frequencies, similar to those that play a vital part in ‘activity silent’ models of working memory. Thus, despite the lack of a cortical organization the avian associative pallium can create LFP signatures reminiscent of those observed in primates. This points towards a critical cognitive function of oscillatory dynamics evolved through convergence in species capable of complex cognition.

## Highlights

• Despite different brain organization, crows and monkeys share oscillatory fingerprints.
• Neural networks required for higher cognition evolved in parallel.
• Contemporary mammalian models of working memory are applicable to birds.

## Graphical Abstract

**Figure:**
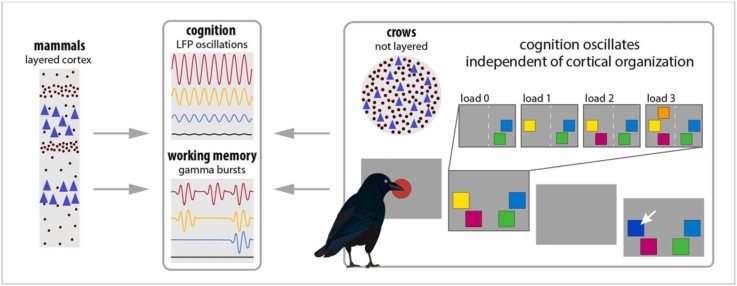

## 1 Introduction

To perform the computations underlying complex cognition, the neuronal ensembles of our brains must be coordinated, otherwise, the chatter of a billion neurons may produce only noise (Lisman, 1997, Miller et al., 2018, Naud and Sprekeler, 2018). Notably, the spiking of individual neurons follows a tight temporal organization that results in regular patterns of excitation and inhibition. At the network level, these patterns of activity can be observed in fluctuations of electrical local field potentials (LFP) that oscillate at different frequencies (Buzsáki et al., 2013, Buzsáki et al., 2012, Buzsáki and Wang, 2012). These frequencies are commonly clustered into bands, for example, the gamma band of frequencies above 30 Hz. Gamma oscillations are likely generated in the superficial layers of cortex (Bastos et al., 2018, Buffalo et al., 2011, Maier et al., 2010), from perisomatic currents around the similarly oriented pyramidal cell layer, and they arise from feedback inhibition between pyramidal cells and soma targeting parvalbumin-positive inhibitory neurons (Buzsáki et al., 2012, Buzsáki and Wang, 2012, Cardin et al., 2009, Carlén et al., 2012, Traub et al., 1996). Functionally, the gamma band has been suggested to be relevant for inter-regional communication of neuronal populations (Fries, 2015) and to play a key role in executive control (Miller et al., 2018). Thus, understanding these coordinated computations is the key to unlocking a functional model of higher cognition.

A cornerstone of complex cognition is working memory (WM), which enables an animal to actively retain and manipulate a limited amount of information to guide behavior (Baddeley et al., 2021). WM is also particularly well suited to investigate higher cognition from a comparative perspective. It was described almost simultaneously in humans and pigeons (Baddeley and Hitch, 1974, Honig, 1978). Furthermore, birds and mammals show similar WM performance (Balakhonov and Rose, 2017, Gibson et al., 2011). For example, the capacity of WM, the number of individual items that can be maintained simultaneously, is comparable between crows and macaque monkeys (Balakhonov and Rose, 2017). Even single neuron correlates of WM in birds are virtually identical to those in mammals (Ditz and Nieder, 2020, Ditz and Nieder, 2016, Moll and Nieder, 2015, Rinnert et al., 2019, Rose and Colombo, 2005), and we recently found that this also extends to the neurophysiological limits of WM capacity (Buschman et al., 2011, Hahn et al., 2021).

Given the large evolutionary distance between the species, these similarities are likely the result of convergent evolution (Emery and Clayton, 2004, Güntürkün and Bugnyar, 2016), and they are sharply contrasted by prominent anatomical differences. Most notably, birds lack the mammalian separation between grey and white matter along with the highly structured organization of the neocortex (Güntürkün and Bugnyar, 2016, Harris and Shepherd, 2015). While recent data suggest a cortex-like circuitry in sensory regions of the avian pallium, a layered neocortex-like structure is absent in associative avian brain regions that are crucial to WM function (Stacho et al., 2020). This includes the avian equivalent of PFC, the nidopallium caudolaterale (NCL), which shares many defining properties of the PFC, including the dense dopaminergic innervation, multimodal sensory afferents, premotor projections, and neuronal correlates for WM (Güntürkün and Bugnyar, 2016, Herold et al., 2011, Kröner and Güntürkün, 1999, Nieder, 2017, Waldmann and Güntürkün, 1993).

Modern models of WM are heavily influenced by the observation of temporal dynamics in the mammalian PFC. In particular, gamma oscillations are closely associated with WM-related processes (Howard et al., 2003, Kornblith et al., 2016, Lundqvist et al., 2016, Roux et al., 2012, Tallon-Baudry et al., 1998). The highly structured organization of the layered mammalian neocortex is an ideal substrate to generate and investigate such oscillations (Einevoll et al., 2013). Consequently, models of temporal dynamics are almost exclusively built on mammalian data. However, whether these cognitive oscillations *require* the specific layered organization of the cortex is unclear. It has even been argued that oscillations could be an epiphenomenon of the underlying network architecture rather than a functional process in itself (Merker, 2013, Ray and Maunsell, 2015). Therefore, the investigation of LFP in avian associative brain regions, lacking the layered organization of the cortex, offers a unique comparative perspective.

To date, only relatively few studies have investigated modulations of LFP in birds. Most prominently, the optic tectum and neighboring tegmental nuclei show modulation in the gamma range during attention (Goddard et al., 2012, Neuenschwander and Varela, 1993, Sridharan et al., 2011, Sridharan and Knudsen, 2015). Gamma band modulations were further reported in the avian forebrain during birdsong (Brown et al., 2021, Lewandowski and Schmidt, 2011, Spool et al., 2021), and in the avian hippocampal formation in vitro (Dheerendra et al., 2018) and during sleep (van der Meij et al., 2020). However, these observations cannot answer the question of whether oscillations underlie higher cognition since they were either made in the neatly layered optic tectum, were tightly linked to motor behavior, or occurred in sleeping birds.

Thus, descriptions of oscillatory dynamics in the non-layered endbrain of birds that are tied to abstract cognition such as WM are still lacking. Hence, it remains unknown if the single-cell similarities extend to oscillatory population dynamics that underlie higher cognition in mammals, or if birds have such cognition without oscillations. If they existed and played comparable roles in avian and mammalian WM, it would be valuable evidence towards general, cross-species mechanisms supporting higher-order cognition.

## 2 Material and methods

Our animals, experimental setup, behavioral protocol, recording setup, and surgical procedures were previously described in Hahn et al. (2021).

### 2.1 Subjects

We worked with two hand-raised carrion crows (*Corvus corone*), held under identical housing and food protocols as described in Hahn et al. (2021). All experimental procedures and housing conditions were carried out in accordance with the National Institutes of Health Guide for Care and Use of Laboratory Animals and were authorized by the national authority (LANUV).

### 2.2 Experimental setup and head tracking

Our setup consisted of an operant training chamber outfitted with a touchscreen (22’’, ELO 2200L APR, Elo Touch Solutions Inc., CA) and an automatic feeder delivering food reward upon correct pecks on the touchscreen. We used two computer vision cameras (‘Pixy’, CMUcam5, Charmed Labs, Tx) to track the birds’ head position via a mount of two lightweight 3D-printed LEDs that was removed after each experimental session. Head-location was acquired at 50 Hz, and data was smoothed by integrating over 2 frames in Matlab using custom programs on a control PC. Birds were required to ‘hold gaze’ with no more than 2 cm horizontal or vertical displacement, and no more than 20° horizontal or vertical angular rotation. The behavioral protocol was executed by custom code written in Matlab using the Psychophysics (Brainard, 1997) and Biopsychology toolboxes (Rose et al., 2008). Further details about the experimental setup have been reported in Hahn et al. (2021).

### 2.3 Behavioral protocol

**Fig. 1:**
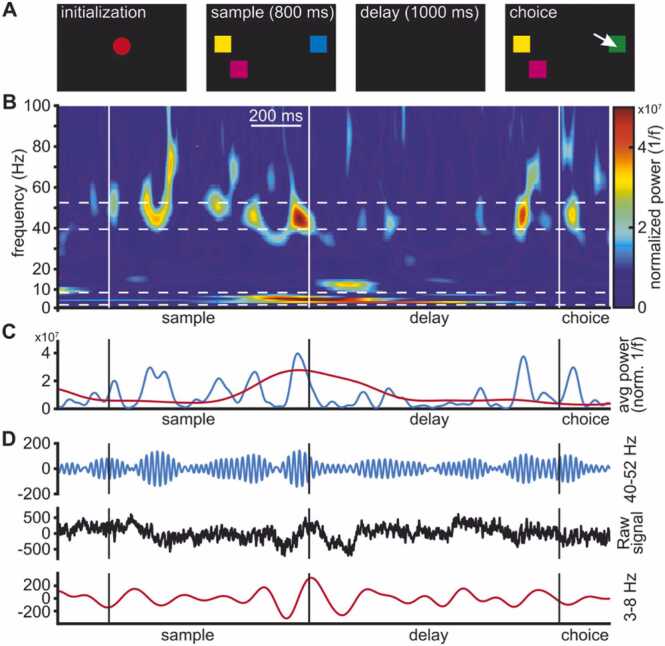
(**A**) Behavioral protocol. After the bird initiated a trial by acquiring and holding head fixation, the sample stimuli (2–5 colored squares distributed so that 0–3 colored squares appeared on each half of the screen) were presented. Birds retained head fixation and maintained color information over a memory delay until the choice stimuli were presented (identical in color and location to those of the sample phase, except for one square that had changed color). Birds then indicated the square that changed color between sample and choice by pecking on it. (**B**) Single-trial example of time-frequency power of LFP. Power was elevated during the transition from sample to delay phase in a band between 3 and 8 Hz. Higher frequencies between 40 and 52 Hz showed recurring increases of power in short bursts during the sample and the delay period, notably also towards the end of the delay. (**C**) Mean power of the selected bands across time (3–8 Hz, red, and 40–52 Hz, blue). The visible peaks correspond to the warmer colors in panel B. (**D**) Raw unfiltered LFP signal (black), and the same signal, band-pass filtered in the range of higher frequencies (blue), and of the lower range frequencies (red). The respective frequency components of the raw signal become visible as their amplitude increased and decreased over time.

We trained the birds to perform a delayed non-match to sample task, previously used to test the performance under different working memory loads in primates (Buschman et al., 2011). The protocol has previously been reported by Hahn et al. (2021). Trials started with the presentation of a red dot centered on the touchscreen (for a maximum of 40 s). Centering of the head in front of the red dot for 160 ms caused the red dot to disappear and a stimulus array of two to five colored squares to appear (Fig. 1A, ‘sample’). The sample was presented for 800 ms, while the animals had to maintain head fixation and center their gaze on the screen. Failure to retain head fixation resulted in an aborted trial. The sample phase was followed by a memory delay of 1000 ms after which the stimulus array reappeared with one color exchanged. The animal indicated which of the colors had changed by pecking the respective square. Correct responses were rewarded probabilistically (BEO special pellets, in 55 % of correct trials, additional 2 s illumination of the food receptacle in 100 % of correct trials). Incorrect responses to colors that had not changed or a failure to respond within 4 s resulted in a brief screen flash and a 10 s timeout. Individual trials were separated by a 2 s inter-trial interval.

The colored squares were presented at six fixed locations on the screen (1–6, Fig. 1A). In each session, one pair of colors was assigned to each of the six locations. Each location had its own distinct pair. These pairs were randomly chosen from a pool of 14 colors (two color combinations were excluded since the animals did not discriminate them equally well during pre-training). Fig. 1A gives an example: the color-change occurred for the middle right where blue (B) is presented during the sample and green (G) during the choice. In this particular session, the middle-right location could thus show either of the following colors during the sample and choice: B-G (shown in Fig. 1A); G-B; G-G; B-B; None-None. On the next session, a new random pair of colors were displayed at this location. The order of presentation of colors within a pair, the target location (where the color change occurred), and the number of stimuli in the array (two to five) were randomized and balanced across trials so that each condition had an equal likelihood to appear. The width of the colored squares was 10 degrees of visual angle (DVA), and squares were placed on the horizontal meridian of the screen and at 45.8 DVA above or below the meridian at a distance of 54 and 55.4 DVA from the center. The binocular visual field of crows is 37.6 DVA (Troscianko et al., 2012). With our arrangement on screen, combined with the head tracking, we ensured that all stimuli appeared only outside of this binocular range.

### 2.4 Surgery

The surgery protocol was identical to the one reported by Hahn et al. (2021). Both animals were chronically implanted with a lightweight head-post to attach a small LED holder during the experiments. Before surgery, animals were deeply anesthetized with ketamine (50 mg/kg) and xylazine (5 mg/kg). Once deeply anesthetized, animals were placed in a stereotaxic frame. After attaching the small head-post with dental acrylic, a microdrive with a multi-channel microelectrode was stereotactically implanted at the craniotomy (Neuronexus Technologies Inc., Ann Arbor MI, DDrive). The electrode was positioned in NCL (AP 5.0, ML 13.0) of the left hemisphere (coordinates for the region based on histological studies on the localization of NCL in crows (Veit and Nieder, 2013)). After the surgery, the crows received analgesics.

### 2.5 Electrophysiological recordings of single-cell activity and LFP

Recordings of neuronal activity (local field potentials and single-cell spiking) were performed using chronically implanted 32-channel microelectrodes (Model A1-32-15 mm, Neuronexus Technologies Inc., Ann Arbor MI). The distance between individual recording sites (electrodes) was 50 µm. The signal was amplified, filtered, and digitized using Intan RHD2000 headstages and a USB-Interface board (Intan Technologies LLC, Los Angeles CA). The system also recorded digital event codes that were sent from the behavioral control PC using a custom IO-device (details available at https://www.ngl.psy.ruhr-uni-bochum.de/ngl/shareware/index.html.en). Before each recording session, the electrodes were advanced manually using the microdrive. Recordings were started 20 min after the advancement, and each recording site was manually checked for neuronal signals (cellular discharges observable on an audio monitor). Signals of analysis of LFP were recorded at a sampling rate of 30 kHz and filtered with a band-pass filter at recording (1 Hz to 7.5 kHz). LFP signals were then further processed by offline down-sampling to 1 kHz. In each session we simultaneously recorded from 32 electrodes, with a combined active zone of 1550 µm. For analysis, we chose to systematically sub-sample a quarter of all electrodes used (i.e., analyzing signals from every fourth electrode, thereby achieving a reduced overlap of signal with 200 µm distance between electrodes). To verify our results, we applied analysis to a second, independent subsample of the electrodes. Qualitative results from this second subsample were comparable. Data of single-cell neuronal activity for analysis of the spiking rate of the neuronal population (Fig. S10) was obtained from our previous study (Hahn et al., 2021).

### 2.6 Processing of LFP results

Prior to extracting frequency power from our signals, we removed possible spike-related traces from the LFP signals using the algorithm of Banaie Boroujeni et al. (2020). We further processed our LFP signals using the FieldTrip open-source software package for Matlab (Oostenveld et al., 2010). We extracted frequency power from the signals using Morlet-wavelet convolution with a Morlet family of 99 frequencies (2–100 Hz), with seven wavelet cycles. We screened all trials for unique trial artifacts centered around 50 Hz during processing. On rare occasions, electrodes had individual trials that showed magnitudes of frequency power up to three magnitudes of power larger than the next biggest power value. We handled such artifacts by restricting data analysis to the 99th percentile of power values on any electrode (i.e., excluding trials from analysis whose power values fell into the top 1% of observed values). During manual curation of results, we nonetheless observed a few electrodes with power levels exceeding their average levels at distinct time points over all frequencies (i.e. power surges not restricted to any specific frequency). Those electrodes (n = 31) were subsequently removed from data analysis altogether.

### 2.7 Statistical testing of power during the trial against baseline power

We tested frequency power during the trial (in load conditions 1–3, at a 1 ms time resolution, across all individual frequencies) against baseline frequency power (i.e., testing the trial phase for a specific load against its baseline during the middle second of the preceding ITI). This preceding ITI was, unlike the following trial period, free of any visual stimulation. We used a permutation approach (Oostenveld et al., 2010) with Monte-Carlo estimates of significance probabilities based on a permutation distribution built from our data (i.e., a data-based null-distribution). We used 1000 permutations, an alpha level of 5 % to determine significance, and an extreme distribution of statistical values to correct for multiple comparisons (i.e., correction was achieved by comparing observed statistical values against the most extreme (minimal and maximal) permutated values). Comparing the observed data to this null-distribution is thereby robust against data-based biases (e.g., non-normally distributed values).

### 2.8 Calculating gamma modulation of individual electrodes

We determined if an electrode was 'gamma modulated' by performing the statistical testing described above for the average power of the 'low gamma band' (33–48 Hz) at load 1, in 100 ms bins with 100 ms steps for the interval beginning at sample start until delay end. We classified electrodes as gamma modulated if two consecutive, non-overlapping bins had been classified as significant.

### 2.9 Statistical testing of power at different loads

We tested the average change in power per added item in five frequency bands (3–7 Hz 'theta', 8–12 Hz 'alpha', 13–19 Hz 'beta', 33–48 Hz 'low gamma', and 83–98 Hz 'high gamma') in bins of 100 ms with a step size of 100 ms. To do so we first calculated the average power within each frequency band and bin, then normalized the average power of each electrode relative to its load 1 condition (i.e., so that power at load 1 was 1 and powers at load 2 and 3 were relative to that), and finally calculated the average between the differences of load 1 and load 2, and load 2 and load 3 (Eq. (1)).

(1) $${P\mathrm{ower}\Delta }_{\mathrm{item}}=\;\frac{\Delta_{\mathrm{load}1,\;\mathrm{load}2}+\;\Delta_{\mathrm{load}2,\;\mathrm{load}3}}{2}$$

We tested if ${\mathrm{Power}\Delta }_{\mathrm{item}}$ was significant by performing a t-test of each individual value against the null-hypothesis that it was non-different from 0 and corrected for multiple comparisons using the Bonferroni method (i.e., $\alpha_{\mathrm{crit}.}$ = 0.0013). We calculated the effect size of the load effect quantified by ${\mathrm{Power}\Delta }_{\mathrm{item}}$ by performing a repeated measures ANOVA (measurements for each electrode at loads 1–3 respectively) over all electrodes and calculating the effect size (ω²) for all individual bins (Eq. (2)).

(2) $$\omega^{2}=\;\frac{{\mathrm{SS}}_{\mathrm{effect}}-\left({\mathrm{df}}_{\mathrm{load}}\right)*\left({\mathrm{MS}}_{\mathrm{error}}\right)}{{\mathrm{SS}}_{\mathrm{total}}+{\mathrm{MS}}_{\mathrm{error}}}$$

### 2.10 Model comparison for location information

To investigate if LFP power contained information about the location of presented stimuli, we performed a comparison of generalized linear models (GLM) applying the method of Kornblith et al. (2016), for comparability of results. We compared a 'full model', containing nested load and location information, to two 'reduced models' where we removed location information about the ipsilateral or contralateral locations and replaced the respective position indicators with their sum. Each model was calculated assuming a normal distribution and its canonical 'identity' link function ($f\left(µ\right)=µ$). For comparison, we also assumed a gamma distribution together with its canonical link function ($f\left(µ\right)=\frac{1}{µ}$). Results of both approaches were similar, but model parameters indicated that the assumption of gamma distribution did not fit all electrodes' data, whereas the normal assumption did. We, therefore, decided to report the results of the normal models. The full model was a GLM with frequency-band power as response variable and the six possible locations as predictors. Each of the six predictors was therefore encoded as either 0 (no color at the location) or 1 (color at the location). For the reduced model we replaced three of the location indicators (either those for the contralateral locations 4–6 or those for the ipsilateral locations 1–3) by their cumulative load (i.e., 0–3). The reduced models thereby lacked information about the respective locations, which, if they were informative about the LFP power, would reduce the model fit (quantified by $R_{\mathrm{adj}.}^{2}$). The difference between the model fits (i.e., ${\Delta R}_{\mathrm{adj}.}^{2}$) then indicates how much information was contained by the respective side’s locations. We calculated this model comparison for six 400 ms bins, with a step size of 400 ms, starting 400 ms before sample onset and ending 200 ms after choice onset. We calculated if ${\Delta R}_{\mathrm{adj}.}^{2}$ was significant in a particular bin by comparing ${\Delta R}_{\mathrm{adj}.}^{2}$ to a null distribution ${\Delta R}_{\mathrm{adj}.\mathrm{Null}}^{2}$ generated from the data by permutation of the data labels prior to performing the model comparison 1000 times. ${\Delta R}_{\mathrm{adj}.}^{2}$ was considered significant if it was bigger than 99.17 % of permutated ${\Delta R}_{\mathrm{adj}.\mathrm{Null}}^{2}$ values (i.e., at an alpha level of 5%, after Bonferroni correction for multiple comparisons).

### 2.11 Calculating burst rates

Burst rates of the individual frequency bands were calculated by detecting threshold crossings of power. Frequency-band power qualifying as burst activity was defined as a power crossing a threshold of mean + 1.5 * SD for at least two consecutive cycles (periods) of the bands center frequency. The mean was calculated over the 10 preceding trials to avoid condition specific systematic changes in power to affect burst detection (i.e., the mean to threshold trial t was calculated from trials t-10 to t-1, similar to Lundqvist et al. (2018b)). For example: to classify an increase in power as a burst in the low gamma band, power had to exceed threshold levels for $2*\frac{1}{\mathrm{centerfrequency}}=2*\frac{1}{45\mathrm{Hz}}=44\mathrm{ms}$. We performed this analysis with a sliding window starting at the start of the sample phase and ending with the end of the delay. We also applied more conservative thresholds for burst events (mean +2 * SD and mean + 3 * SD, and up to three cycles). The results of these more stringent bursting criteria were qualitatively the same as for our initial threshold (Figs. S6 and S7).

To further ensure that our burst counts were driven by task conditions and did not occur randomly, we used the method of Feingold et al. (2015), comparing burst rate of a phase randomized signal to our original signal. For this we calculated the Fourier spectrum of our signal and assigned to each frequency a phase randomly drawn from a uniform distribution of values between -π and π. We then performed a conjugate symmetric inverse Fourier transform on the phase randomized data, resulting in a ‘synthetic signal’ without a complex component. This results in preserved overall power distribution, but power being shuffled in time. We then performed our burst analysis on this synthetic signal to calculate an expected null-distribution of burst rates under randomized conditions. Burst rates resulting from the actual dataset were then compared to the randomized dataset. This gave us an indication to what degree bursts occurring during the trial were driven by ongoing processing, over a random occurrence. To additionally quantify this, we calculated the coefficient of variation of each channel within each analyzed frequency band and compared it between the original data to the surrogate data. This gave us an estimate for the overall variance occurring in both data (Fig. S8).

## 3 Results

### 3.1 Behavior

To investigate LFP dynamics in the avian brain during a complex form of cognition, we trained two crows on a multi-item working memory task, previously used for probing WM capacity in crows and primates (Balakhonov and Rose, 2017, Buschman et al., 2011). On each trial, the crows were presented with a variable number of colored squares that they had to retain over a memory delay. Subsequently, the colors reappeared, and the birds indicated with a single peck which of the squares now had a different color (Fig. 1A). The performance of the crows was load-dependent, gradually declining with higher loads. We analyzed performances based on the number of colored squares present on the side on which the color changed (i.e., ipsilateral to change, in contrast to contralateral to change referring to the number of squares on the side without a change). Median performances for ipsilateral item load of one, two, and three were 95.88 %, 78.31 %, and 58.21 %, respectively. This result is very similar to the performance reported in monkeys in the same task (Buschman et al., 2011) and has been discussed in detail in a previous study (Balakhonov and Rose, 2017).

### 3.2 LFP in the endbrain of crows is task modulated

To investigate if WM modulates oscillations in a comparable way in crows as in primates, we analyzed LFP recorded throughout NCL from a total of 336 electrodes. We performed spectral decomposition of the recorded signal using Morlet-wavelet convolution, after removing neuronal spiking artifacts and 50 Hz line noise (see Section 2 for details). LFP power was affected throughout the time course of a trial in a frequency-dependent manner. To facilitate the comparison to results obtained in primates, we subdivided frequencies into the commonly recognized LFP bands, i.e., ‘theta’, ‘alpha’, ‘beta’, and ‘gamma’ (Miller et al., 2018).

We observed modulation of LFP power in a frequency band (40–52 Hz) of the gamma range during the sample phase and delay phase, as well as high levels of power in a 3–8 Hz frequency band toward the end of the sample phase (Fig. 1B and C). This was also observed in the raw signal trace, most prominently in the sample and towards the end of the delay, when the individual frequency components contributed most to the composite signal (indicated by higher frequency amplitudes in Fig. 1D).

### 3.3 Gamma power modulation is present throughout NCL

**Fig. 2:**
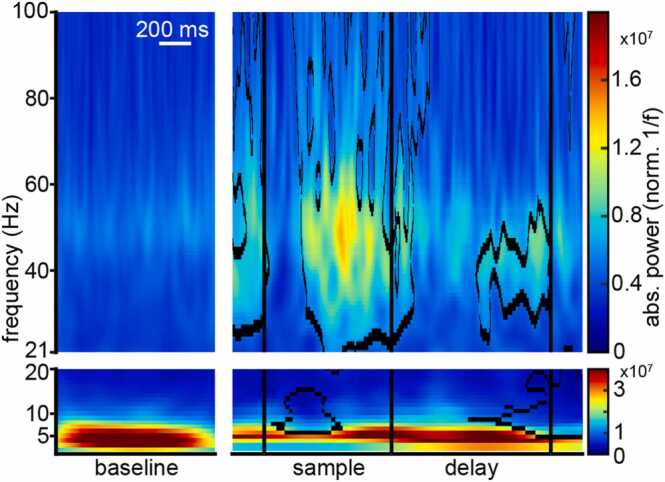
Average time-frequency power of LFP of a single electrode of a single session during the baseline period (1 s during the middle of the inter-trial-interval) and during the trial period. The duration of the pre-sample period was variable dependent on behavior, it could therefore not be used as baseline, and it contains motion and stimulus-viewing. In the sample phase an increase in gamma power, and a decrease in alpha/beta power is detectable. Outlined areas indicate power values significantly different from baseline. Higher and lower frequencies were split to highlight their respective power range that scales with $\frac{1}{f}$. See Supplementary section 1 for more details.

Were specific frequency bands consistently affected by the ongoing cognitive task? We tested trial averaged LFP power during the trial against stable baseline power (see Section 2 for details). Averaging across trials revealed two frequency bands of particular interest (Fig. 2, example electrode). Power in the lower frequency band (2–20 Hz) was significantly suppressed during the early sample and at the end of the delay (Fig. 2, bottom). The higher-frequency band (> 20 Hz) was significantly elevated relative to baseline during the late sample and towards the end of the delay phase (Fig. 2, top; see Fig. S1 for statistical results and further details). Elevated power in the gamma range at the single-trial level was consistent across trials but showed some temporal variance (Fig. S2). Thus, modulations of LFP generated in NCL had narrow and well-defined frequency bands that reflected the different task phases.

Because the gamma frequency range was most affected by our task, we focused on electrodes that showed modulations in that range. The peaks in power in the high gamma frequency bands were the most prominent observation at the level of individual electrodes. Was this a general effect throughout the extent of NCL or was it localized only at specific electrodes, as has been observed in monkey PFC (Lundqvist et al., 2016)? We determined if our electrodes could be classified into ‘gamma-modulated’ and ‘non-modulated’ sites, by calculating significance of a low (33–48 Hz) and high gamma (83–98 Hz) band during the sample phase for each of our electrodes (see Section 2 for details). We found that power in the low gamma band was significantly modulated at 81.64 % (n = 249) of electrodes (76.72 %, n = 234, for high gamma). The electrodes without significant gamma modulation came from recordings obtained at locations within individual sessions (i.e., sessions in which there was no gamma modulation detected at any site), i.e., gamma modulation was either present or absent at the sites of any given recording session. There was also no systematic difference in the recordings obtained throughout the dorso-ventral extent of the NCL. We examined the overall modulations of recorded LFP power from all electrodes with significant gamma modulation.

**Fig. 3:**
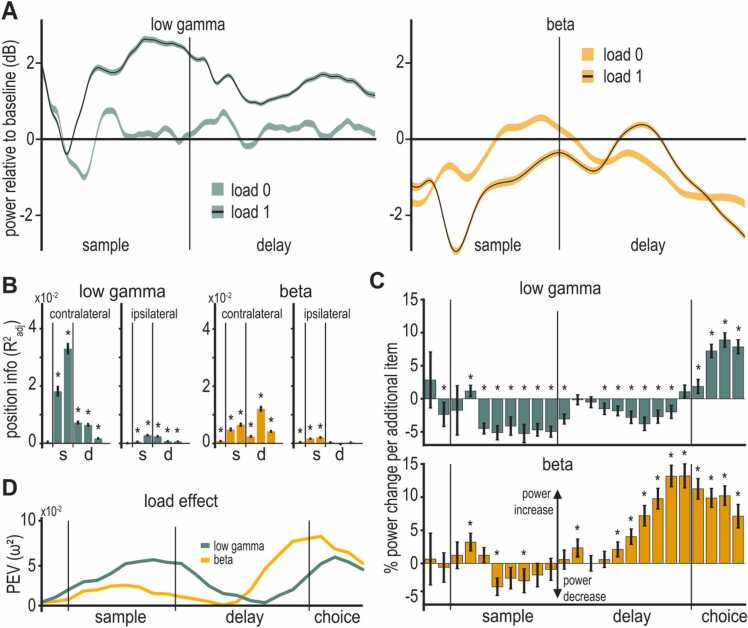
(**A**) LFP in the gamma (top) and beta (bottom) are modulated by working memory. At load 0 no stimuli were presented contralateral to the electrode, at load 1 a single contralateral stimulus was presented during the sample period. Lines indicate the mean; shaded areas indicate the standard error of the mean. (**B**) Position information (mean $\Delta R_{\mathrm{adj}.}^{2}$) contained in average power of the low gamma and beta band (400 ms bins). Power of the low gamma and of the beta frequency band contained information about the contralateral positions of stimuli, in contrast information about the positions of the ipsilateral stimuli was much smaller. Position information for low gamma frequencies was more pronounced during the sample phase than during the delay phase. Error bars indicate the standard error of the mean. Stars indicate significance at the Bonferroni corrected alpha level (α = 0.0083; refer to Fig. S3 for other frequency bands). (**C**) Average change in power per added item (100 ms bins). The low gamma frequency band (33–48 Hz) shows a reduction of power with every added item throughout the sample delay phase but gains power with every added item in the choice phase. The beta frequency band (13–19 Hz) shows a consistent increase in power with every added item throughout sample and delay phase, notably peaking towards the end of the delay. Error bars indicate the standard error of the mean. (**D**) Quantification of the load effect depicted in (C), as percent explained variance by factor power (ω^2^).

The sampled average signal showed that the task phases strongly affected the LFP. Both low gamma frequencies (33–48 Hz ‘low gamma’) and beta band frequencies (13–19 Hz ‘beta’) showed a distinct modulation by the task. The low gamma band was shortly suppressed after the sample onset, followed by an increase in power towards the end of the sample phase (Fig. 3A left). In the memory delay phase, power of these frequencies remained at an elevated level (relative to baseline) and ramped up towards the end of the delay leading up to the choice. Beta frequencies initially showed strong suppression of power during the early sample phase (Fig. 3A right) and returned to baseline levels toward the late sample and early delay. Power was again suppressed towards the end of the delay phase, leading up to the choice.

### 3.4 Gamma modulation reflected working memory processing

The described modulations in power have so far been linked to the processing of the WM task, divided into the processing of presented memory items (during sample), their maintenance (during the delay), and in anticipation of the upcoming change detection (towards the end of the delay). We investigated if our WM task caused further modulation of LFP that reflected cognitive processing of relevant stimulus dimensions by analyzing if power of these bands contained information about the location of the presented items and if the number of items affected power.

To estimate information about positions, we applied the method of Kornblith et al. (2016), performing model comparisons of generalized linear models. Positions were localized relative to electrodes as either ipsilateral (i.e., on the same side as the implanted hemisphere) or as contralateral (opposite side of the hemisphere). For all electrodes that had significant gamma modulation, we derived position information needed to solve the task for the locations by quantifying the difference of model fits (${\Delta R}_{\mathrm{adj}.}^{2}$) in six 400 ms intervals (pre-sample; early/late sample; early/mid/late delay, see Section 2.10 for more details).

**Fig. 4:**
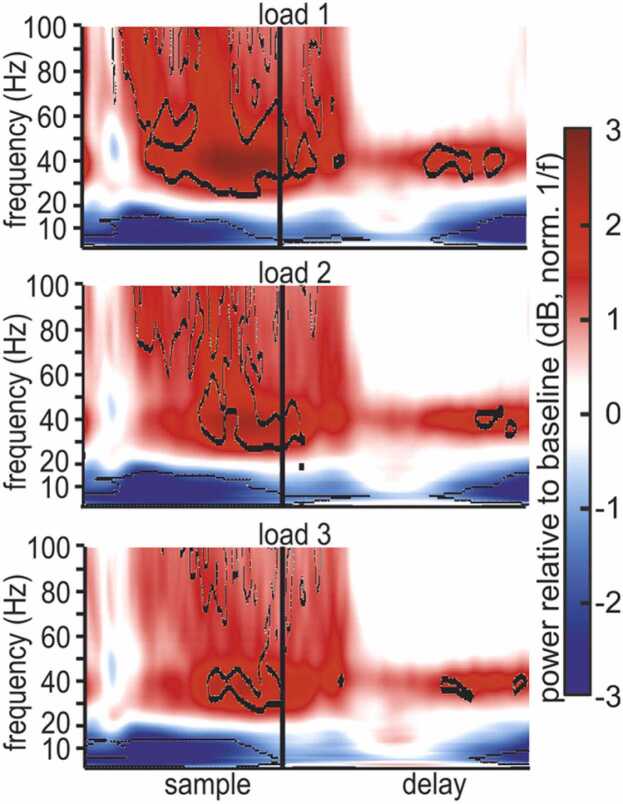
WM load affected the time-frequency power of LFP. Average power of all electrodes with significant gamma band modulation, relative to baseline (in decibel), for load 1–3. Lower frequencies show a general suppression of power, relative to baseline, while higher frequencies show a general increase in power. The tree panels depict different WM-load (number of items contralateral to the recording electrode). Outlined areas indicate significant differences from baseline.

In general, power contained information about the (task relevant) locations of presented squares (Fig. 4B). This information was most prominently present during the sample phase. We found that low gamma power had significant position information during the sample for the contralateral side of the screen (early and late sample, mean (± standard error of the mean (SEM)): 0.0182 (± 0.0019), F(1,1247) = 379.83, p < 0.0001, ω^2^ = 0.2327 and 0.0330 (± 0.0020), F(1,1247) = 1063.8, p < 0.0001, ω^2^ = 0.4597, respectively). Beta band power contained a significant amount of information during the sample (early and late sample, mean (± SEM): 0.0049 (± 0.0007), F(1,1247) = 200.02, p < 0.0001, ω^2^ = 0.1374 and 0.0065 (± 0.0008), F(1,1247) = 299.36, p < 0.0001, ω^2^ = 0.1928, respectively) and notable information during the delay (mid delay, mean (± SEM), 0.0121 (± 0.0011), F(1,1247) = 543.44, p < 0.0001, ω^2^ = 0.2993).

Other frequency bands (3–7 Hz ‘theta’, 8–12 Hz ‘alpha’, and 83–98 Hz ‘high gamma’) also contained information about the contralateral position during the sample phase and delay phases (Fig. S3). However, these frequency bands had much less information compared to the low gamma band (see Supplementary section 2). None of the frequency bands had meaningful information about the ipsilateral locations (refer to Tables S1 and S2 for a detailed overview). This location information contained in LFP power indicates involvement in processing the spatial component of the task, as binding each color to a location was necessary for localizing the change detection.

### 3.5 Working memory load modulated gamma

The major manipulation affecting cognitive processing in our task was the number of squares the birds had to memorize as it determined the load of WM. We considered three load conditions (‘loads’). Because power contained information only for contralateral locations, we analyzed load effects for the number of squares presented in the visual hemifield contralateral to the recording electrode. Trials in which only one square was presented during the sample (Fig. 1A) were considered to have ‘load 1’ (irrespective of the number of squares on the other side of the screen). Following this logic, trials with two or three presented colors were considered ‘load 2’ and ‘load 3’, respectively. To understand how LFP-power was modulated by WM-load, we again compared the power of all gamma-modulated electrodes during the sample and memory delay phases to baseline power during the inter-trial interval. When comparing power across the different loads, the local maximum of power in the low gamma band appeared to be modulated, with higher loads reducing average power (Fig. 4, Fig. S4). Similarly, the power in the lower bands appeared to be affected by load. To better quantify the load effect, we tested its effect on power in the five major frequency bands introduced above (we focus on the low gamma and the beta band that were prominently affected by the overall task, refer to Fig. S5 for the other frequency bands). The mean power in the respective frequency band, across all channels with significant gamma power modulation in load 1 trials, was compared by calculating the average change in power per added item. Power in the low gamma band decreased as load increased during sample and delay but reversed this modulation during the subsequent choice phase (Fig. 3C). The beta band showed the opposite effect of load, with power generally increasing at higher loads. We further quantified the magnitude of the load effect by calculating the effect size (PEV, ω^2^) of the LFP differences for different loads over time (see Section 2 for details). The influence of load on low-gamma power was largest towards the end of the sample (power decreased with load) and in the choice phase (power increased with load). The strongest beta power load modulation started appearing during the middle of the delay phase (power increased with load), peaking at the end of the delay (Fig. 3D, refer to Table S3 for numerical values). This means that LFP power was substantially affected by both the locations of the presented stimuli and by the WM load. Therefore, LFP processes seem to be tightly linked to ongoing cognitive processing of the WM task, during both sample encoding of memory items and their subsequent maintenance during the delay.

### 3.6 Beta and Gamma appear in bursts

**Fig. 5:**
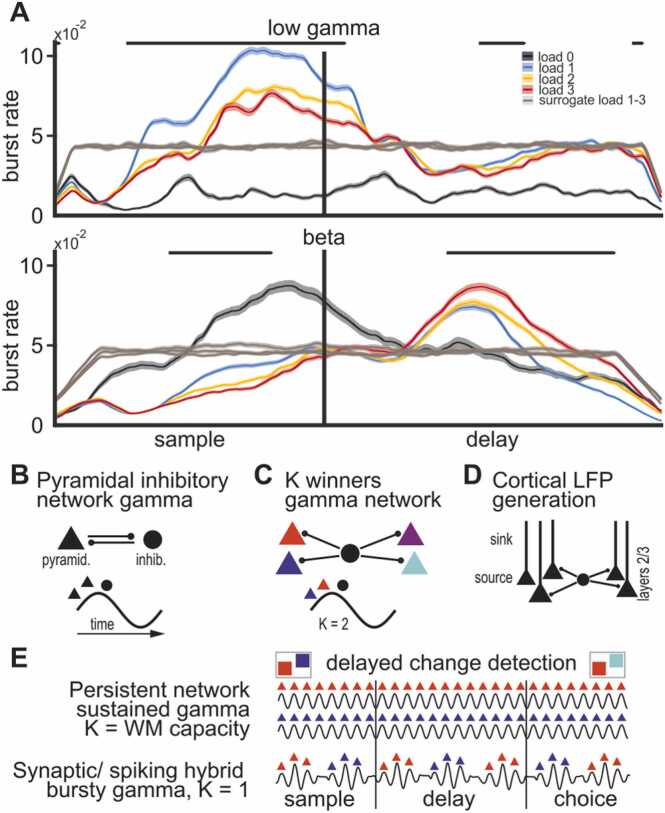
(**A**) Trial burst rate of low gamma and beta frequency bands during the trial at gamma modulated sites. Burst rate of low gamma strongly increases towards the end of the sample phase, while beta has peak burst rate in the middle of the delay. Load modulation occurs with higher loads decreasing burst rate in the sample but increasing burst rate towards the end of the delay. Lines depict the mean, shaded areas indicate the standard error of the mean, black bars indicate consecutive significance between loads 1–3 (p < 0.05) over 2 cycles of the bands center frequency. In brown, burst rates of the respective frequency band, for load 1–3, calculated from the randomized surrogate signal. (**B**) Schematic generation of mammalian pyramidal inhibitory network gamma (PING). Gamma oscillation is generated in a cycle when excitatory pyramidal cells first become active, exciting inhibitory parvalbumin positive interneurons that provide dense, short-lasting feedback inhibition. The inhibition briefly shuts down the pyramidal cells to terminate the cycle. (**C**) Implementation of a winner-take-all dynamic. If several pyramidal populations (colored triangles) are connected to the same inhibitory population (black circle), the gamma generating feedback inhibition can implement a K winners take all dynamic where only the K most excited populations will spike before the feedback inhibition deactivates all populations. For example, the earlier spiking of blue and red in each cycle results in K = 2. (**D**) Cortical layer organization facilitate gamma oscillation. Many similarly aligned pyramidal cells receive rhythmic, peri-somatic inhibition. The pyramidal cells are thought to act as aligned dipoles with the source close to the somas and the sink in the apical dendrites, creating an extracellular field. The gamma in cortical LFPs is thus generated in the superficial layers of cortex. Crow NCL lacks this layered anatomical organization. (**E**) Two different networks solving a 2-item delay change detection task. The two colored squares can be retained either by selective, persistent activity (top) in a network where gamma implements a K = WM capacity winner-take-all algorithm, or alternatively, in a network relying both on intermittent spiking and synaptic mechanisms with K = 1. In the latter, since K = 1, the two memory representations take turn being active and silent resulting in bursting gamma. In the silent periods, information is retained in synaptic changes rather than sustained spiking.

An additional observation we made was that power modulations in the low gamma band appeared as bursts throughout sample and delay phase (Fig. 1B). In a study in which monkeys performed a sequential version of our task (Lundqvist et al., 2016), increases in gamma power were found to originate from sparse and temporarily defined ‘bursts’ of power. We tested if the increase in gamma power was due to individual bursts by investigating the potential burst events. We detected bursts as temporal intervals in which power crossed a set threshold (mean + 1.5 * SD) for a set amount of time (two cycles of the center frequency of the respective band, indicating a lasting deflection of power). To ensure that our burst counts were driven by task conditions and did not occur randomly, we also applied more conservative thresholds (up to 3 * SD above mean for 3 cycles). Results remained qualitatively the same (Figs. S6 and S7). Finally, we used the method of Feingold et al. (2015), comparing burst rates of a surrogate signal to our original signal. The surrogate signal retained the power distribution, but which was randomly shuffled in time due to randomization of the phase of frequency components (see Section 2.11 for details). Burst rates in our data were substantially higher than those of the synthetic data (Figs. 5A and S5, Table S6 for statistical results) and had a broader range of temporal fluctuations (i.e., bursts) as measured by the coefficient of variation (CV, Fig. S8).

We calculated burst rates over time (i.e., the observed rate of bursts at any given time in a trial, see Section 2 for details). Burst rate in the low gamma band increased throughout the sample phase, peaking in the late sample phase (load 1, mean ± SEM, 0.1044 ± 0.0015 at 619 ms), before gradually reducing throughout the delay (Fig. 5A top, Table S4, Fig. S5 for alpha and high gamma). Notably, the burst rate increased again during the latest part of the delay. These burst rates were also load-dependent in two directions depending on the task phase. During the sample phase, the burst rate significantly decreased with load, while during the late delay phase, the burst rate increased with load (Fig. 5A, refer to Table S5 for statistical values). The beta band had peak burst rate during a trough of gamma bursting during the delay (load 3, mean ± SEM, 0.0871 ± 0.0034 at 1262 ms; refer to Figs. S5 and S10 for the other frequency bands and for a comparison of bursts to population spiking rate). The phase and load-dependent rates of low gamma and beta bursts correlated with processing demands of WM for encoding during the sample, maintenance during the delay, and preparation for decoding towards the end of the delay and choice phase. The average length of a burst event (duration measured as the number of cycles of the center frequency), of any frequency band, did not meaningfully change depending on the load. While nominally significant for some comparisons (based on an ANOVA with factor load, Fig. S9), the difference between the loads were so small in absolute terms (< 1 % of cycle length) and inconsistent with regard to the effect direction, that we did not consider it to be relevant. This interpretation was also backed by a subsequent calculation of the effect size which resulted in very small values for each of the significant ANOVAs (all ω^2^ ≤ 0.001). The stability in burst length, across different working memory loads, further supports the notion that random noise fluctuations were not artificially inflating the burst rate. If this had been the case the load dependent increase in burst rate should have also affected burst length.

However, there was a noticeable difference between frequency bands. The average cycle length of burst events for the alpha band was lowest at 2.2344 and increased with the frequency band up to 2.9082 at high gamma. This means high-gamma bursts were ≈ 30 % longer than alpha bursts (relative to their respective cycle length). This general increase in cycle length was also significant (F(3,91288) = 1995, p < 0.0001, ω^2^ = 0.0615).

## 4 Discussion

We observed cognitively modulated oscillations of LFP in the NCL of carrion crows performing a WM task. Oscillations occurred in a narrow gamma band and in the beta band. This data shares many similarities with those observed in monkey PFC. While these results are consistent with behavioral and single-cell observations, they are remarkable given that WM of birds and monkeys have diverging neuronal architectures that evolved independently over the last 320 million years (Benton and Donoghue, 2007).

### 4.1 Cognitively modulated gamma

Gamma range increases in power might have been correlated with motor preparation. While we cannot fully exclude this possibility, several aspects of the task and our analysis diminish the possible role of specific motor (preparation) signals.

First, in addition to tracking head position, we also tracked an angular component to ensure that each eye only saw half of the screen (i.e., the left or right hemifield). This angle dimension also restricted head movements throughout sample and delay. Any movement would have been considerably effortful, for example a parallel movement that did not change the angle of the head. Further, the birds did not know which location would change its color, i.e., they were not able to plan for a peck at any particular location. Color changes happened in the left or right hemifield in a randomized fashion, so that preliminary movement to either side would not have been a favorable strategy. Furthermore, load within one hemifield was independent of the load in the other hemifield (with the exception that their sum always was between 2 and 5). We used this feature in analysis, with our load depending only on the contralateral hemifield (i.e., contralateral to implanted hemisphere). This means that for the highest load of 3 the ipsilateral hemifield will always have had fewer colored squares (0–2), which should then have triggered a movement towards that side that counterbalanced possible strategies such as moving the head towards the side with the smaller or bigger load.

Finally, when we analyzed the LFP for information about location of colored squares (an analysis independent of load) we only found information for the contralateral side, and none for the ipsilateral side (Fig. 3B, Fig. S3). Had the LFP been governed by motor preparation we would have expected to find either information about both hemifields (Supplementary section 2, (Brincat et al., 2021, Husband and Shimizu, 2001)).

Complementing this is the presence of gamma bursts, i.e. on individual trials power did not just steadily increase towards the end of the delay. We therefore will discuss our findings with relation to what we call the cognitive (i.e., non-motor-related) components of the task.

### 4.2 Non-cortical local field potentials

Two major phenomena of our results need to be considered with regard to avian brain: the generation of the rhythm itself and the anatomy needed to pick up the signal in the LFPs.

First, it is believed that similarly oriented dendrites are needed to create dipoles that constructively contribute to the LFPs, such that oscillations in neural activity also are manifested in the LFPs (Fig. 5D, Einevoll et al., 2013). The laminar and columnar organization of the mammalian cortex, with similarly aligned pyramidal cells, is thought to produce extracellular electrical fields that facilitate the observation of rhythmic population activity (Fig. 5B; (Buzsáki et al., 2012; Einevoll et al., 2013)). Gamma oscillations in birds have first been reported in the optic tectum of pigeons (Neuenschwander and Varela, 1993) and barn owls (Sridharan et al., 2011), a midbrain structure that like the mammalian neocortex displays a separation between grey and white matter and organization into highly structured layers (Güntürkün et al., 2020). However, the associative pallium of birds lacks this structure entirely (Güntürkün and Bugnyar, 2016), and the mosaic-like arrangement of fiber patches in NCL (Stacho et al., 2020) differs substantially from the highly structured, layered organization of the PFC. Therefore, our results indicate that the assumption that a (cortical) layering of dendrites is required for oscillations, is not valid. This is in line with recent other observations of modulated gamma in the (non-layered) telencephalon of birds: in the song system of singing zebra finches (Brown et al., 2021, Lewandowski and Schmidt, 2011) and in the hippocampus of sleeping zebra finches (van der Meij et al., 2020). Our results now show that such gamma oscillations occur in a modulated fashion during ongoing higher cognitive functions like working memory.

Second, to generate oscillations themselves, (cortical) models typically rely on feedback loops between excitatory and inhibitory neurons (in particular for gamma). Thus, there is likely some convergent evolution at play with a similar feedback-loop in NCL.

Functionally involved in such gamma oscillations are excitatory cell types, homologous to mammalian excitatory neurons (including parvalbumin positive neurons), which are part of neuronal circuitry that can be optogenetically induced to produce broad range gamma oscillations ((Spool et al., 2021), Fig. 5B). These neurons were found in higher associative parts of the zebra finch pallium, adjacent to NCL (Spool et al., 2021).

Recent advances in comparative neuroanatomy have shown that the caudal nidopallium of crows has undergone substantial differentiation comparable to the differentiation of PFC in primates (Eugen et al., 2020). Therefore, it is possible that the comparable cognitive abilities between primates and crows are based on similar specializations of associative endbrain regions. Unfortunately, a detailed characterization of the microanatomy of different cell types in the NCL of songbirds (in particular in crows) is lacking, and the region as a whole appears to have a complex neuronal architecture, in contrast to more established non-associative regions of the bird brain (Stacho et al., 2020, Eugen et al., 2020).

From a functional perspective, based on the similarities of the observations of gamma in the avian optic tectum, it has been suggested that gamma rhythms play an essential role in information processing and are thus evolutionary conserved (Sridharan and Knudsen, 2015). We now demonstrate that the NCL of crows also shows gamma modulation of the LFP linked to ongoing cognition in the form of WM. This is despite the anatomical differences between the layered PFC and nuclear NCL, in terms of the architecture of the telencephalon at this mesoscale.

Therefore, the firmly established equivalency of avian NCL to mammalian PFC, both functionally (Nieder, 2017) and through its macro anatomy (Güntürkün and Bugnyar, 2016), also holds for its LFP dynamics. This expands our knowledge about how higher cognition (WM) arises in birds, i.e. following the same oscillatory dynamics observed in mammals.

### 4.3 Gamma modulation related to WM

Remarkably, the telencephalic LFP power dynamics in the gamma frequency range is observed across species in a similar fashion: it was elevated during stimulus encoding, contained information about stimulus location, reduced during the early delay, and ramping up towards the end of the delay (Kornblith et al., 2016, Lundqvist et al., 2016).

The observation that gamma oscillations have similar cognitive correlates in crows as in mammals, despite key anatomical differences, could point towards a key functional advantage of rhythmic population activity. This argument was previously made based on the conserved temporal properties across vastly different mammalian brain sizes (Buzsáki et al., 2013). Cortical gamma is thought to implement a winner-take-all algorithm (Fig. 5C) that simultaneously promotes selective neuronal activity without runaway excitation due to divisive normalization (Fries, 2015, Lundqvist et al., 2010). Earlier analysis of changes in spiking with WM load suggested that there is such normalization of spike rates in avian NCL (Hahn et al., 2021) and modeling findings demonstrated that the same neural architecture with feedback inhibition generated both normalization and gamma oscillations (Lundqvist et al., 2010). Taken together this suggests that gamma oscillations are a manifestation of normalization across species and that crow gamma could have a similar role in selection and normalization despite being implemented on a different neural substrate.

We also report that avian gamma is ‘bursty’ rather than a continuous and prolonged increase in power. The smooth elevation during stimulus encoding and the smooth increase during the end of the delay were visible only in the trial averages, at the single-trial level it was only elevated above baseline in brief bursts. Such bursts of gamma have also been observed in human and non-human primate cortex (Kucewicz et al., 2017, Lundqvist et al., 2016, Lundqvist et al., 2018b). They provide support for models in which WM information is retained by a combination of spiking and synaptic mechanisms (Fig. 5E; (Lundqvist et al., 2011; Mongillo et al., 2008; Sandberg et al., 2003)). The role of the bursts may be to facilitate reliable synaptic transmission (Lisman, 1997) and to leave a plastic synaptic mark of WM at the synapse (Miller et al., 2018). This and other related findings have motivated models of WM in which retention can be achieved by ‘activity silent’ mechanisms, i.e., synaptic plasticity following bursts of spiking (Lundqvist et al., 2018a, Miller et al., 2018, Sreenivasan and D’Esposito, 2019). However, there is an ongoing debate over these models and the more classical model of WM retention through observable sustained spiking (Constantinidis et al., 2018, Wang, 2021).

In addition to gamma oscillations, we also observed lower frequency oscillations (4–25 Hz). Similar to alpha/beta oscillations in primates, these largely showed the opposite behavior as the gamma oscillations over time (elevated when gamma was suppressed and vice versa). In cortical networks, alpha/beta oscillations are thought to play an inhibitory role and suppress gamma and the associated processing of sensory information (Händel et al., 2011, Jensen and Mazaheri, 2010, Lundqvist et al., 2016). Gamma band activity, in contrast, is associated with active encoding and decoding of WM information, e.g., when information has to enter WM, or when it is retrieved (Lundqvist et al., 2016, Roux et al., 2012, Sederberg et al., 2003). Thus, during these gamma active phases, the neuronal networks are plastic. Alpha/beta band activity is associated with retention (e.g., during the delay) that safeguards encoded information against perturbation. Our data are largely in line with these ideas, although we also observed some deviations from such mammalian data and model-predictions as outlined above.

### 4.4 Deviations from mammalian models

Despite these striking similarities in the overall modulation of oscillatory activity by task epochs between birds and mammals, we also observed key deviations, in particular for load-dependent effects: despite gamma increasing during WM-encoding (load 1 vs. load 0), it subsequently decreased with load. Similarly, power of the beta band generally decreased during the delay, but additional items increased power (Fig. 3C). This is in stark contrast to studies from human and non-human primates in which gamma increases monotonically with load (Howard et al., 2003, Kornblith et al., 2016, Lundqvist et al., 2016, Meltzer et al., 2008, Roux et al., 2012), and beta decreases monotonically with load (Lundqvist et al., 2016, Kornblith et al., 2016). All models (persistent and activity silent models alike), as far as we are aware, assume that there should be increased activity with load. The finding that gamma decreases with load during encoding is really interesting and puzzling as it poses a challenge to virtually all WM models (computational and primate models alike). The strongest prediction from activity silent models is that activity should be transient or intermittent. This is supported by the bursty nature of the power changes that speaks against persistent models. Further, in the memory delay/retention period gamma bursting increased with load as one would expect from the activity silent model (and here also spiking increased with load in contrast to encoding period so it is not just the LFPs that demonstrate this pattern). From a modeling perspective, a load dependent reduction of bursts could potentially be explained by an increase of simultaneously active populations as load increases. Each population codes for distinct items. Due to the lack of columnar alignment, they could potentially cancel out each other’s contribution to the measured field when more than one is active (in contrast to the cortical alignment, Fig. 5D). However, the positive correlation between load and gamma at the end of the delay and in the choice period could speak against an anatomical explanation for this cross-species discrepancy. It should also be noted that single-neuron spiking only showed a load-dependent effect towards the end of the delay (where it increased with load, similar to mammals), suggesting there are cross-species differences in the population activity, particularly at encoding and not only in the measured LFP. This poses a challenge to existing models of working memory that tend to assume increased cognitive load is supported by increased (or at least not decreasing) population activity (Lundqvist et al., 2011). Another possible explanation could be that the birds processed the memory items differently during the sample and at the end of the delay. Because memory items were presented simultaneously, the birds might have processed them as one during the sample but then shifted to an individual representation during the delay, like cycling through the individual colors one by one. Task-dependent changes, depending on the behavioral relevance in the neural representations of WM items, have been reported in monkeys (Panichello and Buschman, 2021). If there’s a difference between those modes, it might explain why our observations are congruent with those of monkeys from a full sequential version of the task only at the end of the delay (Lundqvist et al., 2016).

We cannot exclude the possibility that some methodological differences (in comparison to monkeys) could have caused our observed deviations. We trained our birds to retain head fixation without restraining them which might have caused effort-related signals that attenuated some effects. Similarly, we did not explicitly control for eye movements. Importantly though, these differences were necessary to attain recordings that would allow our novel LFP analysis of purely task-related cognition. Motor-related activity in particular would have hindered such isolated analysis. Overall, the complex pattern with different load effects during encoding and choice, and non-monotonic changes from load 0 to load 3, points towards intriguing differences in the evolved implementations between mammals and birds. In addition, while gamma and alpha/beta tended to be elevated and suppressed in different parts of the trials, this relationship did not seem as strong as that in primates. For instance, the load effects for gamma and beta bursts went in the same, not opposite, directions as one would expect if they were anti-correlated.

The fact that birds have similar WM capacity, and striking similarities in the neural WM activity, makes these differences more relevant as clues towards what dynamical features are vital to support higher order cognition. Future modeling and avian neurophysiological studies hold significant promise to reveal such principles.

## CRediT authorship contribution statement

**Lukas Alexander Hahn:** Data curation, Formal analysis, Investigation, Methodology, Software, Visualization, Writing – original draft, Writing – review & editing. **Dmitry Balakhonov:** Conceptualization, Data collection, Methodology. **Mikael Lundqvist:** Investigation, Visualization, Writing – review & editing. **Andreas Nieder:** Project administration, Resources, Writing – review & editing. **Jonas Rose:** Conceptualization, Funding acquisition, Methodology, Project administration, Resources, Supervision, Visualization, Writing – review & editing.

## Funding

This work was supported by a Volkswagen Foundation Freigeist Fellowship (93299) awarded to J.R., by Deutsche Forschungsgemeinschaft (DFG, German Research Foundation) Project B13 of the collaborative research center 874 (122679504) and Projektnummer 316803389 - SFB 1280. 10.13039/100010663ERC starting Grant 949131 and 10.13039/501100004359Swedish Research Council starting Grant 2018-04197) awarded to M.L. The funders had no role in study design, data collection, interpretation, or the decision to submit the work for publication.

## Declaration of interests

The authors declare no competing interests.

## Data Availability

Data will be made available on request.

## References

[bib1] Baddeley A.D., Hitch G., Bower G.H. (1974). Psychology of Learning and Motivation.

[bib2] Baddeley A.D., Hitch G., Allen R., Logie R.H., Camos V., Cowan N. (2021). Working Memory State of the Science.

[bib3] Balakhonov D., Rose J. (2017). Crows rival monkeys in cognitive capacity. Sci. Rep..

[bib4] Banaie Boroujeni K., Tiesinga P., Womelsdorf T. (2020). Adaptive spike-artifact removal from local field potentials uncovers prominent beta and gamma band neuronal synchronization. J. Neurosci. Methods.

[bib5] Bastos A.M., Loonis R., Kornblith S., Lundqvist M., Miller E.K. (2018). Laminar recordings in frontal cortex suggest distinct layers for maintenance and control of working memory. Proc. Natl. Acad. Sci. USA.

[bib6] Benton M.J., Donoghue P.C.J. (2007). Paleontological evidence to date the tree of life. Mol. Biol. Evol..

[bib7] Brainard D.H. (1997). The psychophysics toolbox. Spat. Vis..

[bib8] Brincat S.L., Donoghue J.A., Mahnke M.K., Kornblith S., Lundqvist M., Miller E.K. (2021). Interhemispheric transfer of working memories. Neuron.

[bib9] Brown D.E., Chavez J.I., Nguyen D.H., Kadwory A., Voytek B., Arneodo E.M., Gentner T.Q., Gilja V. (2021). Local field potentials in a pre-motor region predict learned vocal sequences. PLoS Comput. Biol..

[bib10] Buffalo E.A., Fries P., Landman R., Buschman T.J., Desimone R. (2011). Laminar differences in gamma and alpha coherence in the ventral stream. Proc. Natl. Acad. Sci. USA.

[bib11] Buschman T.J., Siegel M., Roy J.E., Miller E.K. (2011). Neural substrates of cognitive capacity limitations. Proc. Natl. Acad. Sci. USA.

[bib12] Buzsáki G., Anastassiou C.A., Koch C. (2012). The origin of extracellular fields and currents — EEG, ECoG, LFP and spikes. Nat. Rev. Neurosci..

[bib13] Buzsáki G., Logothetis N., Singer W. (2013). Scaling brain size, keeping timing: evolutionary preservation of brain rhythms. Neuron.

[bib14] Buzsáki G., Wang X.-J. (2012). Mechanisms of gamma oscillations. Annu. Rev. Neurosci..

[bib15] Cardin J.A., Carlén M., Meletis K., Knoblich U., Zhang F., Deisseroth K., Tsai L.-H., Moore C.I. (2009). Driving fast-spiking cells induces gamma rhythm and controls sensory responses. Nature.

[bib16] Carlén M., Meletis K., Siegle J.H., Cardin J.A., Futai K., Vierling-Claassen D., Rühlmann C., Jones S.R., Deisseroth K., Sheng M., Moore C.I., Tsai L.-H. (2012). A critical role for NMDA receptors in parvalbumin interneurons for gamma rhythm induction and behavior. Mol. Psychiatry.

[bib17] Constantinidis C., Funahashi S., Lee D., Murray J.D., Qi X.-L., Wang M., Arnsten A.F.T. (2018). Persistent spiking activity underlies working memory. J. Neurosci..

[bib18] van der Meij J., Rattenborg N.C., Beckers G.J.L. (2020). Divergent neuronal activity patterns in the avian hippocampus and nidopallium. Eur. J. Neurosci..

[bib19] Dheerendra P., Lynch N.M., Crutwell J., Cunningham M.O., Smulders T.V. (2018). In vitro characterization of gamma oscillations in the hippocampal formation of the domestic chick. Eur. J. Neurosci..

[bib20] Ditz H.M., Nieder A. (2020). Format-dependent and format-independent representation of sequential and simultaneous numerosity in the crow endbrain. Nat. Commun..

[bib21] Ditz H.M., Nieder A. (2016). Numerosity representations in crows obey the Weber–Fechner law. Proc. R. Soc. B Biol. Sci..

[bib22] Einevoll G.T., Kayser C., Logothetis N.K., Panzeri S. (2013). Modelling and analysis of local field potentials for studying the function of cortical circuits. Nat. Rev. Neurosci..

[bib23] Emery N.J., Clayton N.S. (2004). The mentality of crows: convergent evolution of intelligence in corvids and apes. Science.

[bib24] Eugen K. von, Tabrik S., Güntürkün O., Ströckens F. (2020). A comparative analysis of the dopaminergic innervation of the executive caudal nidopallium in pigeon, chicken, zebra finch, and carrion crow. J. Comp. Neurol..

[bib25] Feingold J., Gibson D.J., DePasquale B., Graybiel A.M. (2015). Bursts of beta oscillation differentiate postperformance activity in the striatum and motor cortex of monkeys performing movement tasks. Proc. Natl. Acad. Sci. USA.

[bib26] Fries P. (2015). Rhythms for cognition: communication through coherence. Neuron.

[bib27] Gibson B., Wasserman E., Luck S.J. (2011). Qualitative similarities in the visual short-term memory of pigeons and people. Psychon. Bull. Rev..

[bib28] Goddard C.A., Sridharan D., Huguenard J.R., Knudsen E.I. (2012). Gamma oscillations are generated locally in an attention-related midbrain network. Neuron.

[bib29] Güntürkün O., Bugnyar T. (2016). Cognition without cortex. Trends Cogn. Sci..

[bib30] Güntürkün O., Stacho M., Ströckens F., Kaas J.H. (2020). Evolutionary Neuroscience.

[bib31] Hahn L.A., Balakhonov D., Fongaro E., Nieder A., Rose J. (2021). Working memory capacity of crows and monkeys arises from similar neuronal computations. eLife.

[bib32] Händel B.F., Haarmeier T., Jensen O. (2011). Alpha oscillations correlate with the successful inhibition of unattended stimuli. J. Cogn. Neurosci..

[bib33] Harris K.D., Shepherd G.M.G. (2015). The neocortical circuit: themes and variations. Nat. Neurosci..

[bib34] Herold C., Palomero-Gallagher N., Hellmann B., Kroner S., Theiss C., Güntürkün O., Zilles K. (2011). The receptor architecture of the pigeons’ nidopallium caudolaterale: an avian analogue to the mammalian prefrontal cortex. Brain Struct. Funct..

[bib35] Honig W.K., Stewart H.Hulse, Harry Fowler, Werner K.Honig (1978). Cognitive Processes in Animal Cognition.

[bib36] Howard M.W., Rizzuto D.S., Caplan J.B., Madsen J.R., Lisman J., Aschenbrenner-Scheibe R., Schulze-Bonhage A., Kahana M.J. (2003). Gamma oscillations correlate with working memory load in humans. Cereb. Cortex.

[bib37] Husband, S., Shimizu, T., 2001. Evolution of the avian visual system. In: Avian Visual Cognition [On-Line]. Available: 〈Pigeon.Psy.Tufts.Edu/Avc/Husband/〉.

[bib38] Jensen O., Mazaheri A. (2010). Shaping functional architecture by oscillatory alpha activity: gating by inhibition. Front. Hum. Neurosci..

[bib39] Kornblith S., Buschman T.J., Miller E.K. (2016). Stimulus load and oscillatory activity in higher cortex. Cereb. Cortex.

[bib40] Kröner S., Güntürkün O. (1999). Afferent and efferent connections of the caudolateral neostriatum in the pigeon (Columba livia): a retro- and anterograde pathway tracing study. J. Comp. Neurol..

[bib41] Kucewicz M.T., Berry B.M., Kremen V., Brinkmann B.H., Sperling M.R., Jobst B.C., Gross R.E., Lega B., Sheth S.A., Stein J.M., Das S.R., Gorniak R., Stead S.M., Rizzuto D.S., Kahana M.J., Worrell G.A. (2017). Dissecting gamma frequency activity during human memory processing. Brain.

[bib42] Lewandowski B.C., Schmidt M. (2011). Short bouts of vocalization induce long-lasting fast gamma oscillations in a sensorimotor nucleus. J. Neurosci..

[bib43] Lisman J.E. (1997). Bursts as a unit of neural information: making unreliable synapses reliable. Trends Neurosci..

[bib44] Lundqvist M., Compte A., Lansner A. (2010). Bistable, irregular firing and population oscillations in a modular attractor memory network. PLoS Comput. Biol..

[bib45] Lundqvist M., Herman P., Lansner A. (2011). Theta and gamma power increases and alpha/beta power decreases with memory load in an attractor network model. J. Cogn. Neurosci..

[bib46] Lundqvist M., Herman P., Miller E.K. (2018). Working memory: delay activity, yes! Persistent activity? Maybe not. J. Neurosci..

[bib47] Lundqvist M., Herman P., Warden M.R., Brincat S.L., Miller E.K. (2018). Gamma and beta bursts during working memory readout suggest roles in its volitional control. Nat. Commun..

[bib48] Lundqvist M., Rose J., Herman P., Brincat S.L., Buschman T.J., Miller E.K. (2016). Gamma and beta bursts underlie working memory. Neuron.

[bib49] Maier A., Adams G., Aura C., Leopold D. (2010). Distinct superficial and deep laminar domains of activity in the visual cortex during rest and stimulation. Front. Syst. Neurosci..

[bib50] Meltzer J.A., Zaveri H.P., Goncharova I.I., Distasio M.M., Papademetris X., Spencer S.S., Spencer D.D., Constable R.T. (2008). Effects of working memory load on oscillatory power in human intracranial EEG. Cereb. Cortex.

[bib51] Merker B. (2013). Cortical gamma oscillations: the functional key is activation, not cognition. Neurosci. Biobehav. Rev..

[bib52] Miller E.K., Lundqvist M., Bastos A.M. (2018). Working memory 2.0. Neuron.

[bib53] Moll F.W., Nieder A. (2015). Cross-modal associative mnemonic signals in crow endbrain neurons. Curr. Biol..

[bib54] Mongillo G., Barak O., Tsodyks M. (2008). Synaptic theory of working memory. Science.

[bib55] Naud R., Sprekeler H. (2018). Sparse bursts optimize information transmission in a multiplexed neural code. Proc. Natl. Acad. Sci. USA.

[bib56] Neuenschwander S., Varela F.J. (1993). Visually triggered neuronal oscillations in the pigeon: an autocorrelation study of tectal activity. Eur. J. Neurosci..

[bib57] Nieder A. (2017). Inside the corvid brain—probing the physiology of cognition in crows. Curr. Opin. Behav. Sci. Comp. Cogn..

[bib58] Oostenveld R., Fries P., Maris E., Schoffelen J.-M. (2010). FieldTrip: open source software for advanced analysis of MEG, EEG, and invasive electrophysiological data. Comput. Intell. Neurosci..

[bib59] Panichello M.F., Buschman T.J. (2021). Shared mechanisms underlie the control of working memory and attention. Nature.

[bib60] Ray S., Maunsell J.H.R. (2015). Do gamma oscillations play a role in cerebral cortex. Trends Cogn. Sci..

[bib61] Rinnert P., Kirschhock M.E., Nieder A. (2019). Neuronal correlates of spatial working memory in the endbrain of crows. Curr. Biol..

[bib62] Rose J., Colombo M. (2005). Neural correlates of executive control in the avian brain. PLoS Biol..

[bib63] Rose J., Otto T., Dittrich L. (2008). The Biopsychology-Toolbox: a free, open-source Matlab-toolbox for the control of behavioral experiments. J. Neurosci. Methods.

[bib64] Roux F., Wibral M., Mohr H.M., Singer W., Uhlhaas P.J. (2012). Gamma-band activity in human prefrontal cortex codes for the number of relevant items maintained in working memory. J. Neurosci..

[bib65] Sandberg A., Tegnér J., Lansner A. (2003). A working memory model based on fast Hebbian learning. Netw. Comput. Neural Syst..

[bib66] Sederberg P.B., Kahana M.J., Howard M.W., Donner E.J., Madsen J.R. (2003). Theta and gamma oscillations during encoding predict subsequent recall. J. Neurosci..

[bib67] Spool J.A., Macedo-Lima M., Scarpa G., Morohashi Y., Yazaki-Sugiyama Y., Remage-Healey L. (2021). Genetically identified neurons in avian auditory pallium mirror core principles of their mammalian counterparts. Curr. Biol..

[bib68] Sreenivasan K.K., D’Esposito M. (2019). The what, where and how of delay activity. Nat. Rev. Neurosci..

[bib69] Sridharan D., Boahen K., Knudsen E.I. (2011). Space coding by gamma oscillations in the barn owl optic tectum. J. Neurophysiol..

[bib70] Sridharan D., Knudsen E.I. (2015). Gamma oscillations in the midbrain spatial attention network: linking circuits to function. Curr. Opin. Neurobiol. SI: Brain Rhythms Dyn. Coord..

[bib71] Stacho M., Herold C., Rook N., Wagner H., Axer M., Amunts K., Güntürkün O. (2020). A cortex-like canonical circuit in the avian forebrain. Science.

[bib72] Tallon-Baudry C., Bertrand O., Peronnet F., Pernier J. (1998). Induced γ-band activity during the delay of a visual short-term memory task in humans. J. Neurosci..

[bib73] Traub R.D., Whittington M.A., Stanford I.M., Jefferys J.G.R. (1996). A mechanism for generation of long-range synchronous fast oscillations in the cortex. Nature.

[bib74] Troscianko J., von Bayern A.M.P., Chappell J., Rutz C., Martin G.R. (2012). Extreme binocular vision and a straight bill facilitate tool use in New Caledonian crows. Nat. Commun..

[bib75] Veit L., Nieder A. (2013). Abstract rule neurons in the endbrain support intelligent behaviour in corvid songbirds. Nat. Commun..

[bib76] Waldmann C., Güntürkün O. (1993). The dopaminergic innervation of the pigeon caudolateral forebrain – immunocytochemical evidence for a prefrontal cortex in birds. Brain Res..

[bib77] Wang X.-J. (2021). 50 Years of mnemonic persistent activity: quo vadis. Trends Neurosci..

