# Supplementary material for: Oscillations without cortex: Working memory modulates brainwaves in the endbrain of crows
Source: Prog Neurobiol. 2022 Dec;219:102372. doi: 10.1016/j.pneurobio.2022.102372 (PMC9749082; doi:10.1016/j.pneurobio.2022.102372)
Supplement: Supplementary file 1 — Supplementary material [file mmc1.docx]

**Supplementary Material**

**Supplementary section 1**

**Low frequencies in Fig. 2.** Local minimum during the early sample phase occurred for a band between 8 and 20 Hz: avg. Local minimum at 8 Hz, 238 ms: 3.1780*10^6^ ± 3.4119*10^5^). The frequency band between 4 and 20 Hz had minimal power at the end of the delay (avg. local minimum within significant region at 16 Hz, 1723 ms: 2.6861x10^6 ± 3.6926*10^5).

**High frequencies in Fig. 2.** Power peaks of the higher frequencies were clustered in a band centered around 47 Hz during the middle of the sample phase (avg. local maximum of the sample phase (±standard error of the mean (SEM)) within significant region at 47 Hz, 483 ms: 1.4211*10^7^ ± 2.0555*10^6^), and several smaller power peaks occurred throughout the delay phase (Fig. 2).


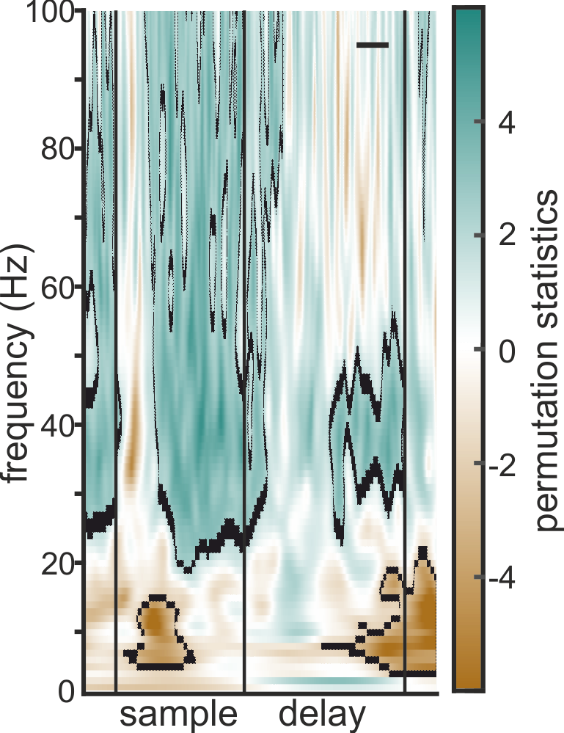


**Figure S1:** *Statistical values of example electrode (Fig. 2). T-values of the significance test of load 1 vs. baseline, axes are identical to those in Fig. 2. Positive values (green) indicate that power was larger than baseline, negative values (brown) indicate that power was smaller than baseline.*

**
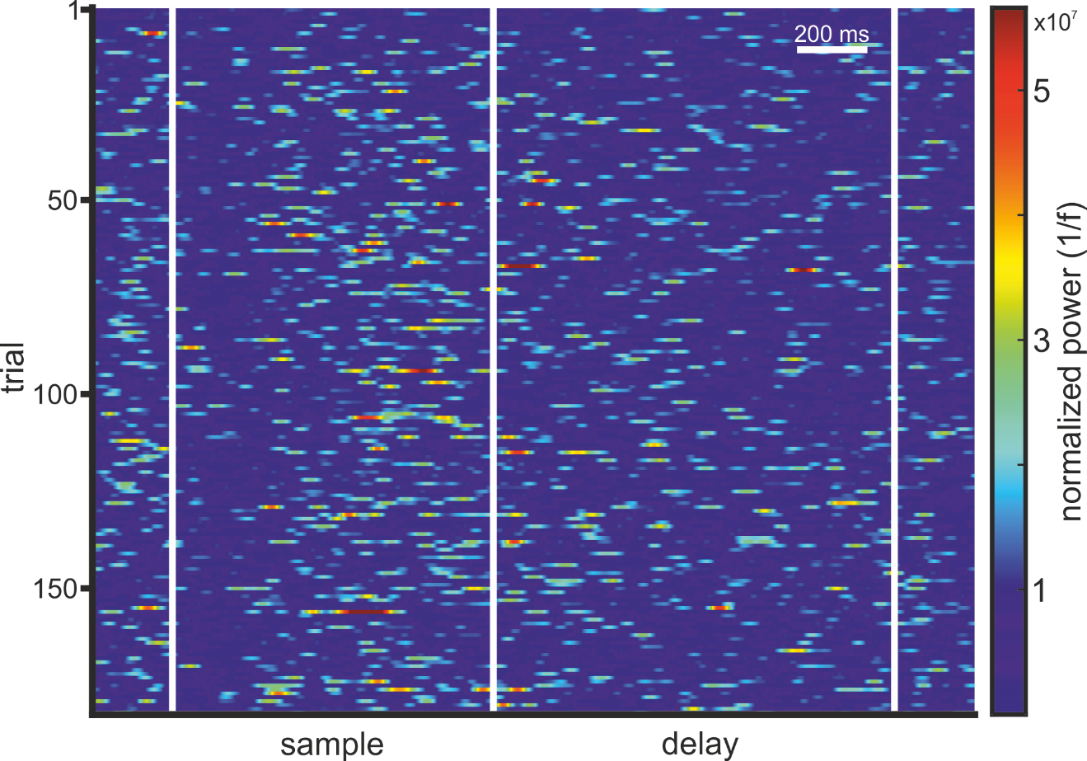
**

**Figure S2:** *Time-frequency-power plot of all trials, recorded from an example electrode (see Fig.1A for a more detailed view of an example trial, and Fig.2 for the average power across all trials). Bursts of power (warmer colors), appear throughout the trial, but are most prevalent towards the end of the sample phase (see also average burst rates in Fig. 5A). Power is limited to the gamma band (41-54 Hz) depicted in Fig.1A.*

**Supplementary section 2**

**Power modulation based on the location of stimuli:** Power of LFP contained information about the location of the colored square, mainly about the contralateral locations but not for the ipsilateral locations. The absolute amount of information contained in LFP power in our data seemed to be slightly higher than that observed in monkeys (Kornblith et al., 2016). Position information was only present during the sample phase, not during the delay, and only for the positions contralateral to the electrode site, this indicates that gamma power plays a part in processing stimulus location unihemispherically. The optic nerve of birds is fully decussated, i.e. information observed by the right eye ends up (via the major visual pathway) exclusively in the left hemisphere (Husband and Shimizu, 2001). We designed our task to make use of this neuroanatomical isolation. Birds had to retain head fixation so that stimuli of the right side of the screen (the side contralateral to electrode implantation) were only visible to the right eye. Our results seem to reflect this manipulation indicating that interhemispheric 'cross talk' did not happen during WM encoding in the sample phase, suggesting independent hemispheric processing. This is in line with results from monkeys (Brincat et al., 2021; Buschman et al., 2011; Kornblith et al., 2016) and the behavioral results of this study (Balakhonov and Rose, 2017).


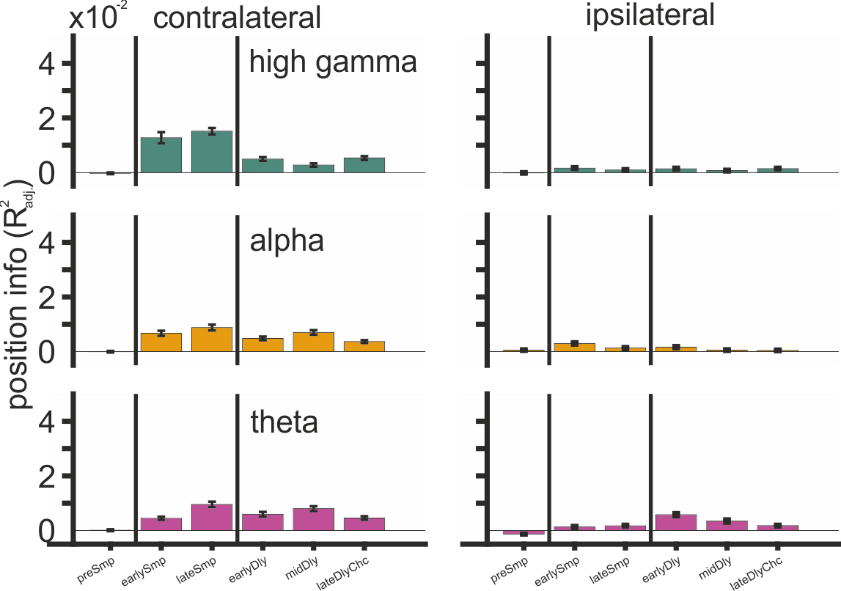


**Figure S3:** *Position information (*$\Delta R_{adj.}^{2}$*) contained in average power of the theta, alpha, and high gamma bands (400 ms bins).*


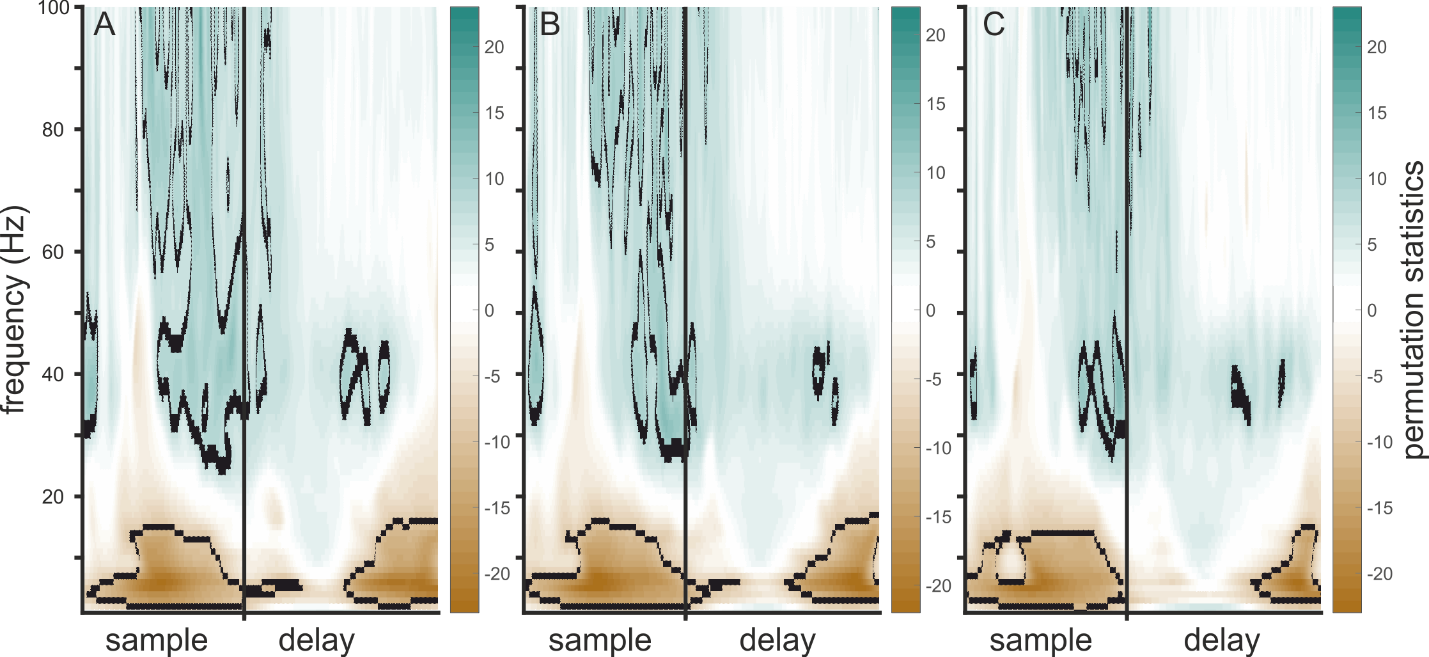


**Figure S4*:*** *Statistical values of all significant electrodes, axes are identical to those in Fig. 4. (A) T-values of the significance test of load 1 vs. baseline. (B) T-values of the significance test of load 2 vs. baseline. (C), T-values of the significance test of load 3 vs. baseline.*

**Supplementary section 3**

Bursts were also present in the alpha and high gamma frequency bands, where bursts occurred during early sample, reducing with load and remaining load-independent at a stable level during late sample, before gradually reducing throughout the delay (Fig. S4, table S4 & S5).


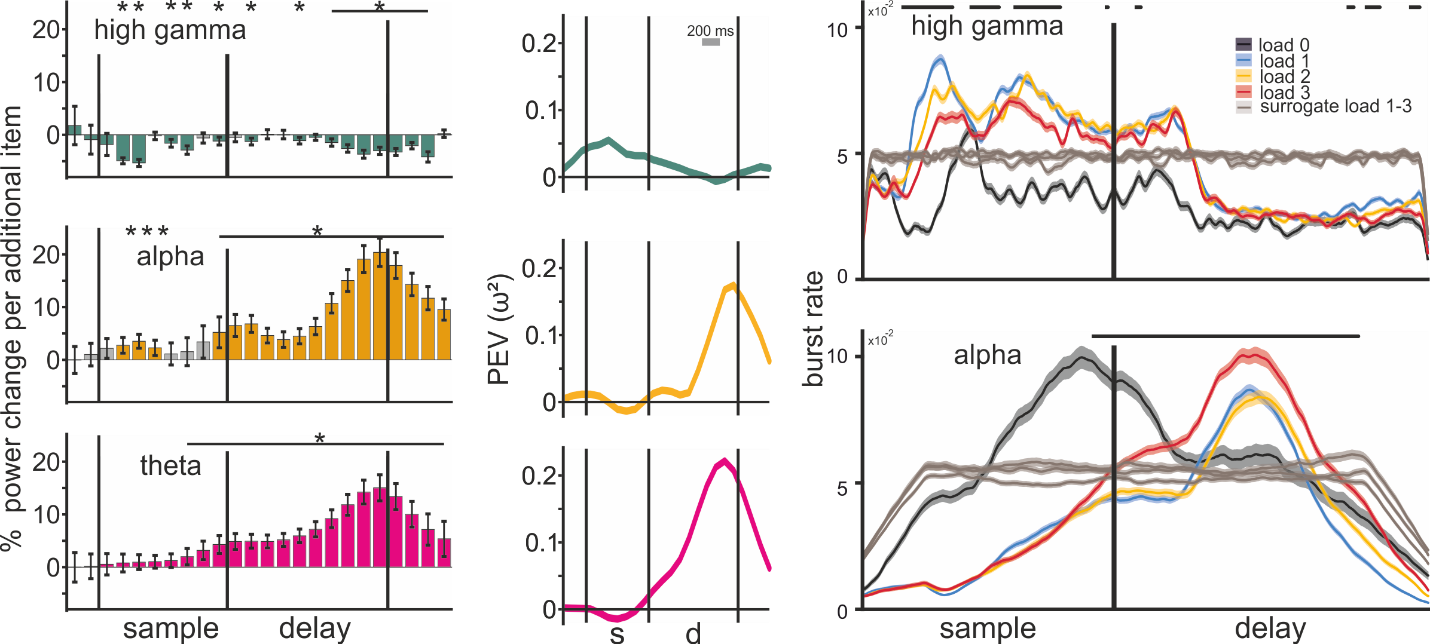


**Figure S5:** *Left column: Average change in power per added item (100 ms bins) for the theta, alpha, and high gamma band. Middle: Quantification of the load effect depicted in the left column, as percent explained variance by factor power (ω²). Right column: Trial burst rate of alpha and high gamma frequency bands during the trial at gamma modulated sites.* *In brown, burst rates of the respective frequency band, for load 1-3, calculated from the randomized surrogate signal. Black bars indicate consecutive significance between loads 1-3 (p < 0.05) over 2 cycles of the bands center frequency.*


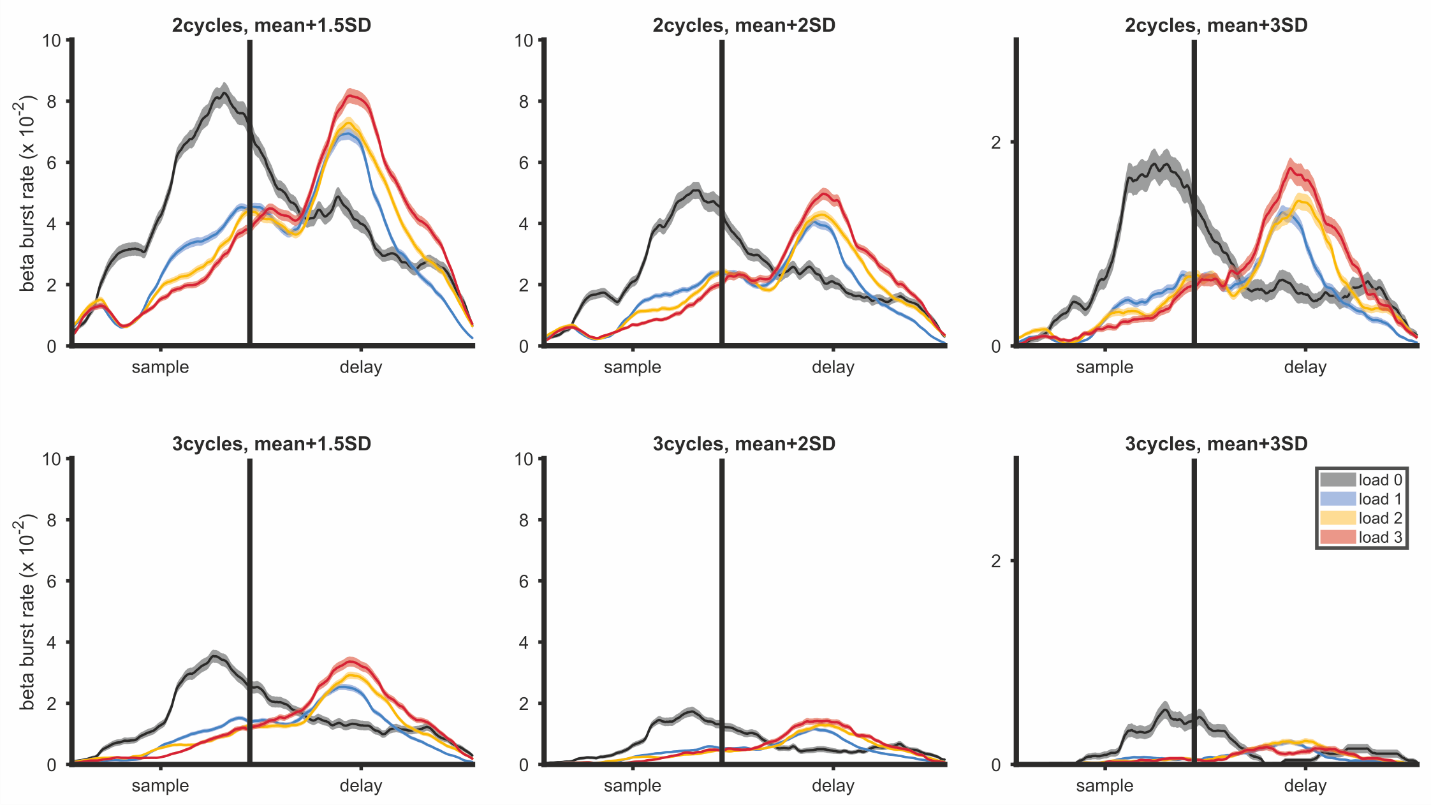


**Figure S6:** *Trial burst rates of beta band for different thresholding parameters. From left to right and top to bottom threshold levels become more conservative (higher threshold and longer crossings). Consequently, the quantity of burst events reduces (mind the different limits on the y-axes). Importantly, the quality of burst events (load effects and general phases of high and low burstiness) remains across threshold levels.*


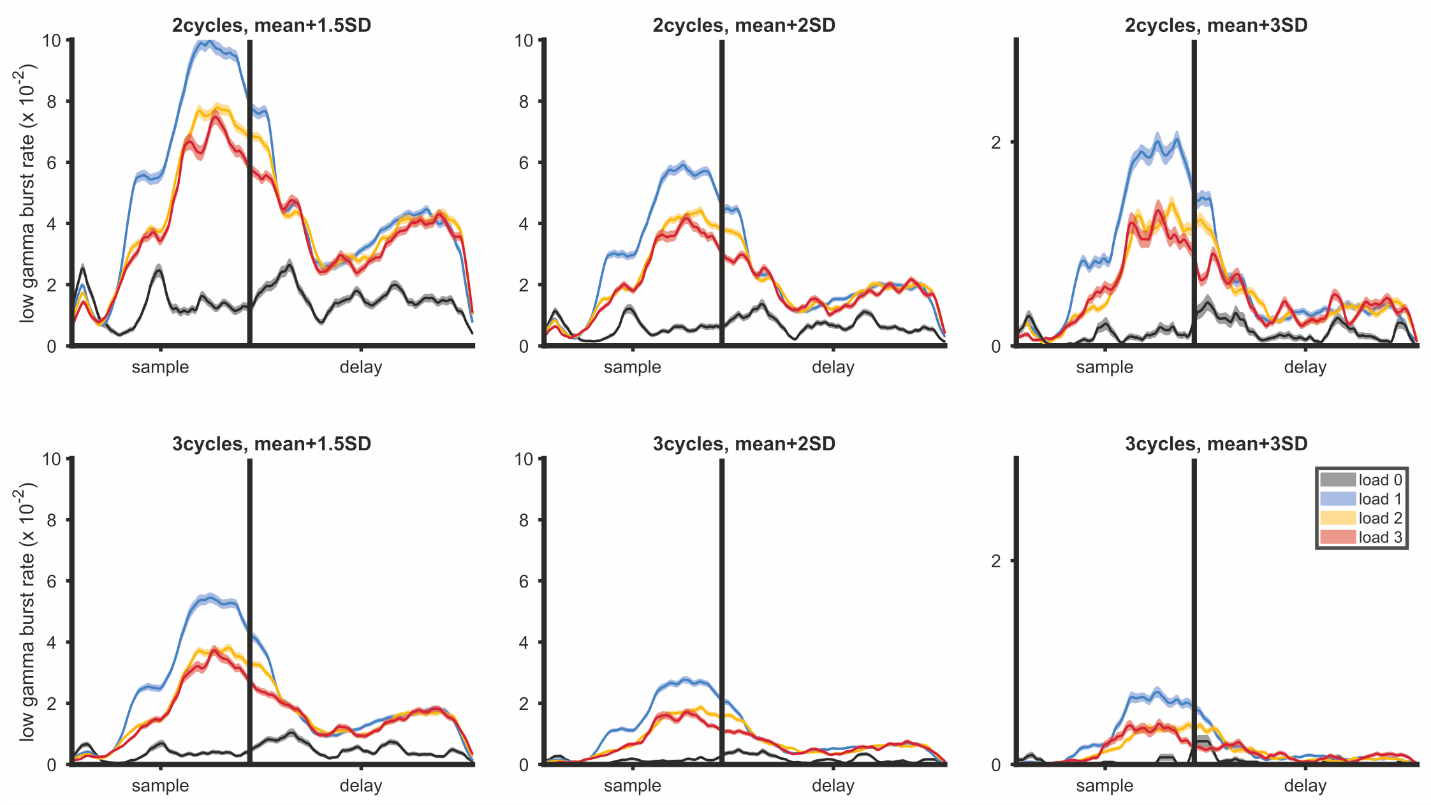


**Figure S7:** *Trial burst rates of low gamma band for different thresholding parameters. From left to right and top to bottom threshold levels become more conservative (higher threshold and longer crossings). Consequently, the quantity of burst events reduces (mind the different limits on the y-axes). Importantly, the quality of burst events (load effects and general phases of high and low burstiness) remains across threshold levels.*


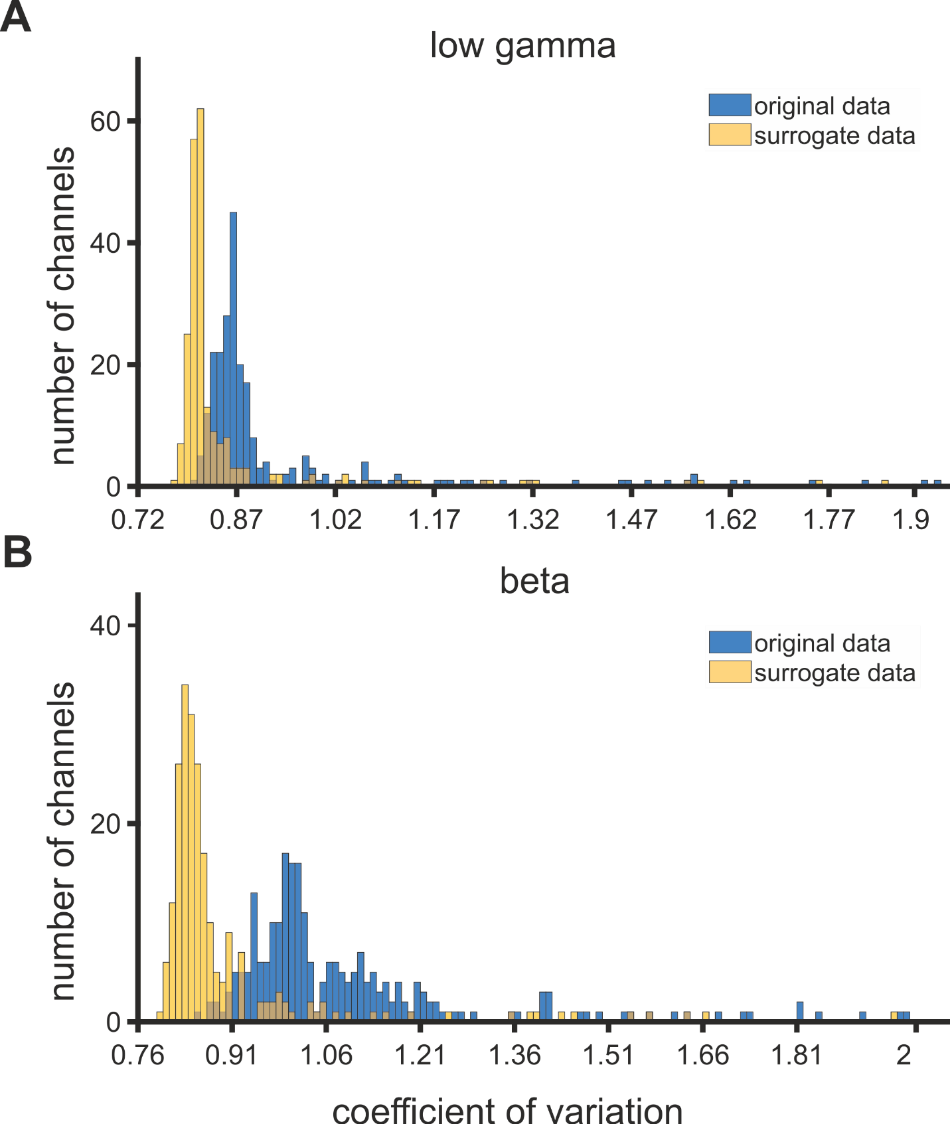


**Figure S8:** *Coefficient of variation (CV) of power in the low gamma band (A) and the beta band (B), for the original data set and for the surrogate data set where phases of the power were randomized prior to power extraction. Power in the original data had substantially more fluctuation in the temporal domain than in surrogate data (indicated by the distribution of CV). CV of surrogate data was significantly smaller (i.e., less bursty) than that of the original data (Wilcoxon rank sum test for low gamma: z = 10.33, p < 0.0001; beta: z = 11.93, p < 0.0001). The distribution of values showed a very long tail towards high values, in particular for the original data, to aid visualization only the lower 95 % of values are being depicted.*

**
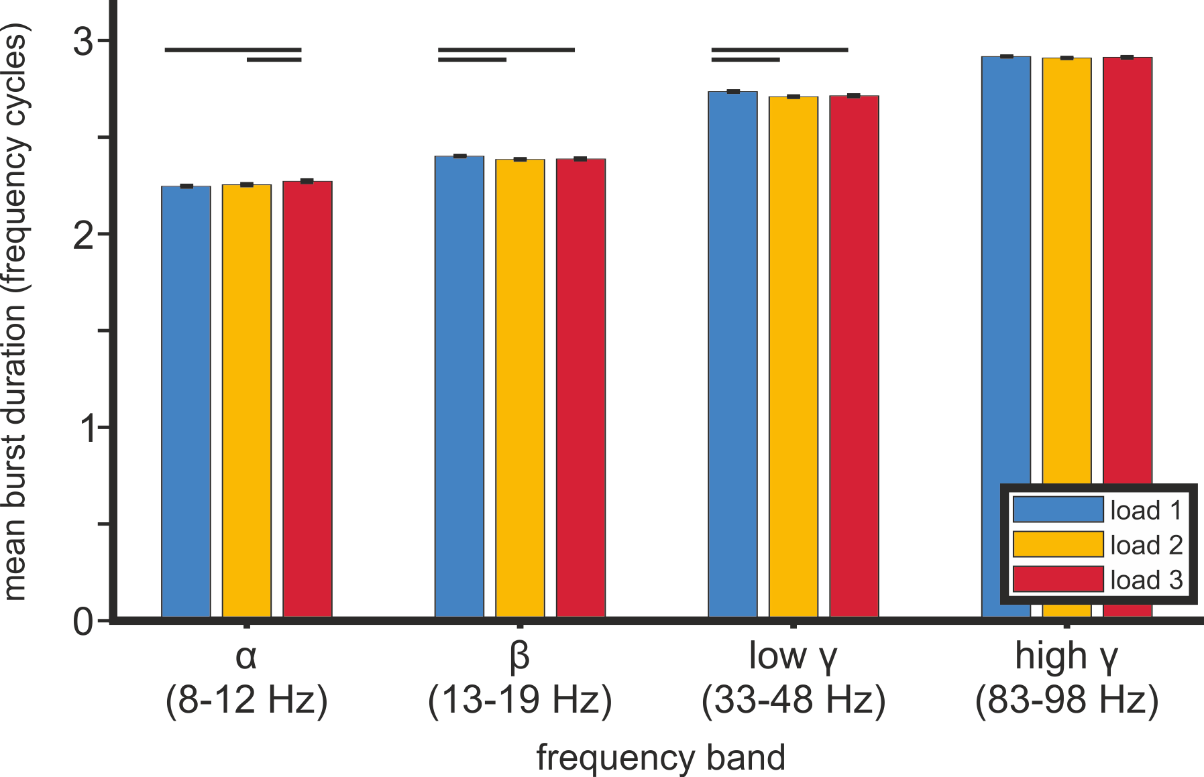
**

**Figure S9:** *Average length of bursts for the different frequency bands and loads. Within each frequency band differences in burst duration are minimal. Burst length increases with frequency.*

**Neuronal spiking of the population.** We also compared the load effect of bursts to the spiking activity of the neurons recorded at the same time. The population of neurons increased its spiking rate during the early sample period and then gradually reduced it throughout the rest of the trial until the choice phase (Fig. S. 5). Load only significantly affected spiking rate towards the end of the delay, with higher loads slightly increasing spiking rate (F(1,2) = 1.3, p = 0.001, ω² = 0.01, posthoc comparisons between load 1/2 and load 2/3, both p < 0.05).


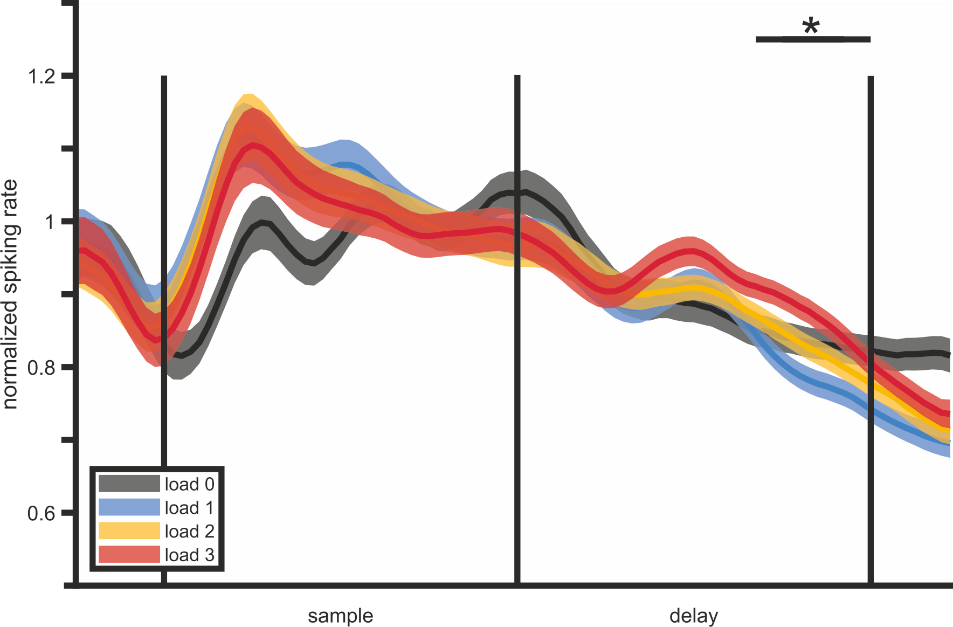


**Figure S10:** *Normalized spiking rate of the neuronal population recorded during the task. The black horizontal bar indicates a significant difference between load conditions.*
