# Supplementary material for: Oscillations without cortex: Working memory modulates brainwaves in the endbrain of crows
Source: Prog Neurobiol. 2022 Dec;219:102372. doi: 10.1016/j.pneurobio.2022.102372 (PMC9749082; doi:10.1016/j.pneurobio.2022.102372)
Supplement: Supplementary file 2 — Supplementary material [file mmc2.pdf]

Table S1: Numerical values of maxima of position information

	average position information contralateral ($R^2_{adj.}$)					
frequency	preSmp	earlySmp	lateSmp	earlyDly	midDly	lateDly
$\theta(3-7Hz)$	0.0001	0.0045	0.0096	0.0059	0.008	0.0046
$\alpha(8-12Hz)$	0	0.0067	0.0088	0.0048	0.007	0.0037
$\beta(13-19Hz)$	0.0008	0.0049	0.0065	0.0025	0.0121	0.0042
$\gamma(33-48Hz)$	0.0004	0.0182	0.033	0.0073	0.0064	0.0017
$\Gamma(83-98Hz)$	-0.0003	0.0127	0.0152	0.0049	0.0028	0.0053
	standard error of the mean (contralateral)					
$\theta(3-7Hz)$	0.0003	0.0006	0.001	0.0009	0.0009	0.0006
$\alpha(8-12Hz)$	0.0003	0.0009	0.0011	0.0007	0.0008	0.0005
$\beta(13-19Hz)$	0.0004	0.0007	0.0008	0.0005	0.0011	0.0005
$\gamma(33-48Hz)$	0.0003	0.0019	0.002	0.0007	0.0007	0.0005
$\Gamma(83-98Hz)$	0.0003	0.0021	0.0012	0.0007	0.0006	0.0007
	effect size of test against null distribution (ω^2)					
$\theta(3-7Hz)$	-0.0006	0.1584	0.2323	0.1274	0.21	0.1384
$\alpha(8-12Hz)$	-0.0006	0.142	0.1811	0.1409	0.1899	0.1307
$\beta(13-19Hz)$	0.0138	0.1374	0.1928	0.0691	0.2993	0.1716
$\gamma(33-48Hz)$	0.0044	0.2327	0.4597	0.2584	0.2159	0.0419
$\Gamma(83-98Hz)$	0.0014	0.1092	0.3477	0.1384	0.063	0.1576
	average position information ipsilateral ($R^2_{adj.}$)					
$\theta(3-7Hz)$	-0.0013	0.0013	0.0017	0.0058	0.0034	0.0018
$\alpha(8-12Hz)$	0.0006	0.003	0.0013	0.0016	0.0005	0.0004
$\beta(13-19Hz)$	0.0005	0.0017	0.0021	0.0004	-0.0001	0.0004
$\gamma(33-48Hz)$	0.0001	0.0006	0.0029	0.0025	0.0008	0.0007
$\Gamma(83-98Hz)$	-0.0001	0.0016	0.0009	0.0014	0.0007	0.0015
	standard error of the mean (ipsilateral)					
$\theta(3-7Hz)$	0.0003	0.0005	0.0005	0.0007	0.0007	0.0004
$\alpha(8-12Hz)$	0.0004	0.0005	0.0004	0.0005	0.0004	0.0004
$\beta(13-19Hz)$	0.0003	0.0004	0.0004	0.0004	0.0003	0.0004
$\gamma(33-48Hz)$	0.0004	0.0004	0.0004	0.0005	0.0004	0.0003
$\Gamma(83-98Hz)$	0.0003	0.0005	0.0004	0.0005	0.0004	0.0004
	effect size of test against null distribution (ω^2)					
$\theta(3-7Hz)$	0.0689	0.0218	0.0399	0.196	0.0736	0.0566
$\alpha(8-12Hz)$	0.0072	0.1012	0.0256	0.0352	0.0055	0.0042
$\beta(13-19Hz)$	0.0092	0.0531	0.0695	0.0033	-0.0005	0.0033
$\gamma(33-48Hz)$	-0.0007	0.0051	0.1166	0.0801	0.0123	0.0125
$\Gamma(83-98Hz)$	-0.0005	0.0376	0.0172	0.025	0.0097	0.038

Table S2:Statistics of position information.

ID	F	df_1	df_2	p
$\theta(3-7Hz)contraPreSmp$	0.3103	1	1247	0.5776
$\theta(3-7Hz)contraEarlySmp$	236.1105	1	1247	< 0.0001
$\theta(3-7Hz)contraLateSmp$	378.9953	1	1247	< 0.0001
$\theta(3-7Hz)contraEarlyDly$	183.325	1	1247	< 0.0001
$\theta(3-7Hz)contraMidDly$	332.9131	1	1247	< 0.0001
$\theta(3-7Hz)contraLateDlyEarlyChc$	201.582	1	1247	< 0.0001
$\theta(3-7Hz)ipsiPreSmp$	93.4328	1	1247	< 0.0001
$\theta(3-7Hz)ipsiEarlySmp$	28.8243	1	1247	< 0.0001
$\theta(3-7Hz)ipsiLateSmp$	52.9692	1	1247	< 0.0001
$\theta(3-7Hz)ipsiEarlyDly$	305.5275	1	1247	< 0.0001
$\theta(3-7Hz)ipsiMidDly$	100.2107	1	1247	< 0.0001
$\theta(3-7Hz)ipsiLateDlyEarlyChc$	75.9505	1	1247	< 0.0001
$\alpha(8-12Hz)contraPreSmp$	0.196	1	1247	0.6581
$\alpha(8-12Hz)contraEarlySmp$	207.6518	1	1247	< 0.0001
$\alpha(8-12Hz)contraLateSmp$	277.1249	1	1247	< 0.0001
$\alpha(8-12Hz)contraEarlyDly$	205.7884	1	1247	< 0.0001
$\alpha(8-12Hz)contraMidDly$	293.8236	1	1247	< 0.0001
$\alpha(8-12Hz)contraLateDlyEarlyChc$	188.7494	1	1247	< 0.0001
$\alpha(8-12Hz)ipsiPreSmp$	10.015	1	1247	0.0016
$\alpha(8-12Hz)ipsiEarlySmp$	141.6304	1	1247	< 0.0001
$\alpha(8-12Hz)ipsiLateSmp$	33.8392	1	1247	< 0.0001
$\alpha(8-12Hz)ipsiEarlyDly$	46.5335	1	1247	< 0.0001
$\alpha(8-12Hz)ipsiMidDly$	7.9221	1	1247	0.005
$\alpha(8-12Hz)ipsiLateDlyEarlyChc$	6.2923	1	1247	0.0123
$\beta(13-19Hz)contraPreSmp$	18.4705	1	1247	< 0.0001
$\beta(13-19Hz)contraEarlySmp$	200.0195	1	1247	< 0.0001
$\beta(13-19Hz)contraLateSmp$	299.3604	1	1247	< 0.0001
$\beta(13-19Hz)contraEarlyDly$	93.7528	1	1247	< 0.0001
$\beta(13-19Hz)contraMidDly$	534.4364	1	1247	< 0.0001
$\beta(13-19Hz)contraLateDlyEarlyChc$	259.699	1	1247	< 0.0001
$\beta(13-19Hz)ipsiPreSmp$	12.6119	1	1247	0.0004
$\beta(13-19Hz)ipsiEarlySmp$	71.0949	1	1247	< 0.0001
$\beta(13-19Hz)ipsiLateSmp$	94.2461	1	1247	< 0.0001
$\beta(13-19Hz)ipsiEarlyDly$	5.0947	1	1247	0.0242
$\beta(13-19Hz)ipsiMidDly$	0.4002	1	1247	0.5271
$\beta(13-19Hz)ipsiLateDlyEarlyChc$	5.1808	1	1247	0.023
$\gamma(33-48Hz)contraPreSmp$	6.4733	1	1247	0.0111
$\gamma(33-48Hz)contraEarlySmp$	379.8323	1	1247	< 0.0001
$\gamma(33-48Hz)contraLateSmp$	1063.827	1	1247	< 0.0001
$\gamma(33-48Hz)contraEarlyDly$	436.2718	1	1247	< 0.0001
$\gamma(33-48Hz)contraMidDly$	344.8669	1	1247	< 0.0001
$\gamma(33-48Hz)contraLateDlyEarlyChc$	55.5925	1	1247	< 0.0001
$\gamma(33-48Hz)ipsiPreSmp$	0.1014	1	1247	0.7502
$\gamma(33-48Hz)ipsiEarlySmp$	7.44	1	1247	0.0065
$\gamma(33-48Hz)ipsiLateSmp$	165.8605	1	1247	< 0.0001
$\gamma(33-48Hz)ipsiEarlyDly$	109.7774	1	1247	< 0.0001
$\gamma(33-48Hz)ipsiMidDly$	16.5156	1	1247	< 0.0001
$\gamma(33-48Hz)ipsiLateDlyEarlyChc$	16.8524	1	1247	< 0.0001

Table 2: continued				
$\Gamma(83-98Hz)contraPreSmp$	2.7801	1	1247	0.0957
$\Gamma(83-98Hz)contraEarlySmp$	154.0675	1	1247	< 0.0001
$\Gamma(83-98Hz)contraLateSmp$	666.6498	1	1247	< 0.0001
$\Gamma(83-98Hz)contraEarlyDly$	201.7003	1	1247	< 0.0001
$\Gamma(83-98Hz)contraMidDly$	85.0001	1	1247	< 0.0001
$\Gamma(83-98Hz)contraLateDlyEarlyChc$	234.6346	1	1247	< 0.0001
$\Gamma(83-98Hz)ipsiPreSmp$	0.3795	1	1247	0.538
$\Gamma(83-98Hz)ipsiEarlySmp$	49.7564	1	1247	< 0.0001
$\Gamma(83-98Hz)ipsiLateSmp$	22.8253	1	1247	< 0.0001
$\Gamma(83-98Hz)ipsiEarlyDly$	32.9647	1	1247	< 0.0001
$\Gamma(83-98Hz)ipsiMidDly$	13.1832	1	1247	0.0003
$\Gamma(83-98Hz)ipsiLateDlyEarlyChc$	50.3882	1	1247	< 0.0001

Table S3: Average change per item, numerical values and statistics.

bin start relative to sample on (ms)	avg. chg. per item ($*10^{-2}$)% ±SEM, test vs. 0; effect size of factor load
$\theta(3-7Hz)$	
-200	-0.0002 ± 0.028, t(288) = 0.01833, p =0.9854; ω^2 = 0.0059
-100	0.0016 ± 0.0235, t(288) = -0.18974, p =0.8496; ω^2 = 0.0032
0	0.0055 ± 0.0202, t(288) = -0.74963, p =0.4541; ω^2 = -0.0017
100	0.008 ± 0.017, t(288) = -1.2945, p =0.1965; ω^2 = -0.0026
200	0.0095 ± 0.014, t(288) = -1.8655, p =0.0631; ω^2 = 0.002
300	0.0103 ± 0.0122, t(288) = -2.3301, p =0.0205; ω^2 = 0.0019
400	0.0124 ± 0.0129, t(288) = -2.6689, p =0.008; ω^2 = -0.0262
500	0.0198 ± 0.0155, t(288) = -3.5227, p =0.0005; ω^2 = -0.0374
600	0.0318 ± 0.0174, t(288) = -5.039, p <0.0001; ω^2 = -0.0126
700	0.0429 ± 0.0169, t(288) = -6.9947, p <0.0001; ω^2 = 0.0209
800	0.0486 ± 0.015, t(288) = -8.9559, p <0.0001; ω^2 = 0.0412
900	0.0492 ± 0.0131, t(288) = -10.3168, p <0.0001; ω^2 = 0.045
1000	0.0487 ± 0.0122, t(288) = -11.0283, p <0.0001; ω^2 = 0.0465
1100	0.0517 ± 0.0122, t(288) = -11.7262, p <0.0001; ω^2 = 0.0584
1200	0.0592 ± 0.0129, t(288) = -12.6267, p <0.0001; ω^2 = 0.0865
1300	0.0718 ± 0.0144, t(288) = -13.7098, p <0.0001; ω^2 = 0.1366
1400	0.0918 ± 0.0168, t(288) = -15.0213, p <0.0001; ω^2 = 0.2025
1500	0.1182 ± 0.0197, t(288) = -16.537, p <0.0001; ω^2 = 0.2471
1600	0.1423 ± 0.0226, t(288) = -17.3278, p <0.0001; ω^2 = 0.2502
1700	0.1503 ± 0.0248, t(288) = -16.7352, p <0.0001; ω^2 = 0.2248
1800	0.1339 ± 0.0245, t(288) = -15.0652, p <0.0001; ω^2 = 0.1833
1900	0.1003 ± 0.0242, t(288) = -11.4106, p <0.0001; ω^2 = 0.1308
2000	0.0714 ± 0.0297, t(288) = -6.6298, p <0.0001; ω^2 = 0.0859
2100	0.0537 ± 0.0334, t(287) = -4.4192, p <0.0001; ω^2 = 0.0529
$\alpha(8-12Hz)$	
-200	-0.0004 ± 0.0257, t(268) = 0.038799, p =0.9691; ω^2 = 0.006
-100	0.01 ± 0.0215, t(268) = -1.2311, p =0.2194; ω^2 = 0.0003
0	0.0217 ± 0.0187, t(269) = -3.0882, p =0.0022; ω^2 = 0.0034
100	0.0276 ± 0.015, t(269) = -4.9084, p <0.0001; ω^2 = 0.0289
200	0.0352 ± 0.0133, t(269) = -7.0544, p <0.0001; ω^2 = 0.0197
300	0.0227 ± 0.0147, t(269) = -4.1294, p <0.0001; ω^2 = 0.0056
400	0.0112 ± 0.0209, t(269) = -1.4248, p =0.1554; ω^2 = -0.0133
500	0.0154 ± 0.0264, t(269) = -1.5565, p =0.1208; ω^2 = -0.0437
600	0.034 ± 0.0305, t(269) = -2.9688, p =0.0033; ω^2 = -0.0223
700	0.0521 ± 0.0292, t(269) = -4.7633, p <0.0001; ω^2 = 0.0062
800	0.065 ± 0.021, t(269) = -8.2519, p <0.0001; ω^2 = 0.0199
900	0.0681 ± 0.016, t(269) = -11.324, p <0.0001; ω^2 = 0.048
1000	0.0461 ± 0.014, t(269) = -8.7594, p <0.0001; ω^2 = 0.0126
1100	0.0384 ± 0.0147, t(269) = -6.98, p <0.0001; ω^2 = 0.0016
1200	0.0448 ± 0.0146, t(269) = -8.1937, p <0.0001; ω^2 = -0.002
1300	0.0631 ± 0.0154, t(269) = -10.8933, p <0.0001; ω^2 = -0.0074
1400	0.1071 ± 0.0184, t(269) = -15.5377, p <0.0001; ω^2 = 0.066
1500	0.1502 ± 0.0205, t(269) = -19.5323, p <0.0001; ω^2 = 0.1728
1600	0.1909 ± 0.0257, t(269) = -19.8154, p <0.0001; ω^2 = 0.1969
1700	0.2037 ± 0.0268, t(269) = -20.2351, p <0.0001; ω^2 = 0.2055
1800	0.1787 ± 0.0246, t(269) = -19.3425, p <0.0001; ω^2 = 0.1967
1900	0.143 ± 0.0213, t(269) = -17.8667, p <0.0001; ω^2 = 0.1
2000	0.1168 ± 0.0221, t(269) = -14.0688, p <0.0001; ω^2 = 0.0719
2100	0.0953 ± 0.0201, t(269) = -12.643, p <0.0001; ω^2 = 0.0656

Table 3: continued	
$\beta(13-19Hz)$	
-200	0.0067 ± 0.0391, t(191) = -0.38236, p =0.7026; ω^2 = 0.0131
-100	-0.0061 ± 0.0221, t(192) = 0.62171, p =0.5349; ω^2 = 0.0147
0	0.0122 ± 0.0201, t(192) = -1.3742, p =0.171; ω^2 = -0.0003
100	0.0323 ± 0.0132, t(192) = -5.5143, p <0.0001; ω^2 = 0.011
200	0.0125 ± 0.0114, t(192) = -2.4815, p =0.0139; ω^2 = 0.0265
300	-0.0354 ± 0.0132, t(192) = 6.0399, p <0.0001; ω^2 = 0.058
400	-0.0225 ± 0.0161, t(192) = 3.1334, p =0.002; ω^2 = 0.0217
500	-0.0262 ± 0.018, t(192) = 3.2814, p =0.0012; ω^2 = 0.0202
600	-0.0175 ± 0.0177, t(192) = 2.2277, p =0.0271; ω^2 = 0.0164
700	-0.0089 ± 0.0167, t(192) = 1.2002, p =0.2315; ω^2 = 0.0154
800	0.0059 ± 0.0145, t(192) = -0.91096, p =0.3635; ω^2 = 0.0232
900	0.0236 ± 0.0133, t(192) = -4.0074, p <0.0001; ω^2 = 0.0184
1000	0.0002 ± 0.0117, t(192) = -0.033248, p =0.9735; ω^2 = 0.0044
1100	0.0061 ± 0.0123, t(192) = -1.1262, p =0.2615; ω^2 = 0.0042
1200	0.0212 ± 0.0113, t(192) = -4.2481, p <0.0001; ω^2 = 0.0007
1300	0.0403 ± 0.0118, t(192) = -7.7319, p <0.0001; ω^2 = 0.0044
1400	0.0721 ± 0.0154, t(192) = -10.5736, p <0.0001; ω^2 = 0.0368
1500	0.0977 ± 0.0156, t(192) = -14.1347, p <0.0001; ω^2 = 0.0725
1600	0.1319 ± 0.0162, t(192) = -18.3303, p <0.0001; ω^2 = 0.118
1700	0.1321 ± 0.0181, t(192) = -16.4302, p <0.0001; ω^2 = 0.0797
1800	0.1126 ± 0.0154, t(192) = -16.4416, p <0.0001; ω^2 = 0.0787
1900	0.0988 ± 0.0143, t(192) = -15.5418, p <0.0001; ω^2 = 0.0513
2000	0.1023 ± 0.0147, t(192) = -15.7297, p <0.0001; ω^2 = 0.085
2100	0.0714 ± 0.0176, t(192) = -9.1154, p <0.0001; ω^2 = 0.0485
$\gamma(33-48Hz)$	
-200	0.0284 ± 0.0424, t(248) = -1.7162, p =0.0874; ω^2 = 0.0084
-100	-0.0237 ± 0.0183, t(248) = 3.3185, p =0.001; ω^2 = 0.016
0	-0.0176 ± 0.0374, t(248) = 1.2047, p =0.2295; ω^2 = 0.0062
100	0.0117 ± 0.0086, t(248) = -3.4988, p =0.0006; ω^2 = 0.0313
200	-0.0451 ± 0.0082, t(248) = 14.0087, p <0.0001; ω^2 = 0.0592
300	-0.0514 ± 0.0107, t(248) = 12.297, p <0.0001; ω^2 = 0.0485
400	-0.0414 ± 0.0138, t(248) = 7.6807, p <0.0001; ω^2 = 0.0244
500	-0.0527 ± 0.0139, t(248) = 9.7, p <0.0001; ω^2 = 0.0453
600	-0.0471 ± 0.0095, t(248) = 12.7011, p <0.0001; ω^2 = 0.0838
700	-0.0497 ± 0.0083, t(248) = 15.2964, p <0.0001; ω^2 = 0.0716
800	-0.0306 ± 0.0076, t(248) = 10.2899, p <0.0001; ω^2 = 0.0584
900	-0.0015 ± 0.0076, t(248) = 0.51818, p =0.6048; ω^2 = 0.0098
1000	-0.0053 ± 0.0084, t(248) = 1.6083, p =0.109; ω^2 = 0.0387
1100	-0.0153 ± 0.0086, t(248) = 4.578, p <0.0001; ω^2 = 0.0262
1200	-0.0184 ± 0.0074, t(248) = 6.3718, p <0.0001; ω^2 = 0.0044
1300	-0.0282 ± 0.0092, t(248) = 7.8115, p <0.0001; ω^2 = 0.0172
1400	-0.0379 ± 0.0102, t(248) = 9.54, p <0.0001; ω^2 = -0.0012
1500	-0.027 ± 0.01, t(248) = 6.9461, p <0.0001; ω^2 = 0.0096
1600	-0.0199 ± 0.0099, t(248) = 5.1705, p <0.0001; ω^2 = 0.0152
1700	0.0107 ± 0.0101, t(248) = -2.6985, p =0.0074; ω^2 = 0.0446
1800	0.0187 ± 0.0107, t(248) = -4.452, p <0.0001; ω^2 = 0.0499
1900	0.0722 ± 0.0101, t(248) = -18.2718, p <0.0001; ω^2 = 0.0711
2000	0.089 ± 0.0108, t(248) = -21.1006, p <0.0001; ω^2 = 0.0799
2100	0.0786 ± 0.0106, t(248) = -18.974, p <0.0001; ω^2 = 0.0525

Table 3: continued	
$\Gamma(83-98Hz)$	
-200	0.0176 ± 0.0364, t(229) = -1.1913, p =0.2348; ω^2 = 0.0056
-100	-0.0091 ± 0.0275, t(233) = 0.82159, p =0.4121; ω^2 = 0.0148
0	-0.0183 ± 0.0211, t(230) = 2.1443, p =0.0331; ω^2 = 0.0103
100	-0.0491 ± 0.0057, t(233) = 21.2992, p <0.0001; ω^2 = 0.0797
200	-0.0532 ± 0.0067, t(233) = 19.596, p <0.0001; ω^2 = 0.0842
300	-0.002 ± 0.0071, t(233) = 0.68699, p =0.4928; ω^2 = 0.0389
400	-0.0159 ± 0.0073, t(233) = 5.3819, p <0.0001; ω^2 = 0.029
500	-0.0288 ± 0.008, t(233) = 8.8849, p <0.0001; ω^2 = 0.0434
600	-0.0059 ± 0.0096, t(233) = 1.536, p =0.1259; ω^2 = 0.0284
700	-0.0118 ± 0.0076, t(233) = 3.8498, p =0.0002; ω^2 = 0.0319
800	-0.0049 ± 0.0083, t(233) = 1.4611, p =0.1453; ω^2 = 0.0272
900	-0.0127 ± 0.0066, t(233) = 4.8156, p <0.0001; ω^2 = 0.0282
1000	0.0011 ± 0.0088, t(233) = -0.30018, p =0.7643; ω^2 = 0.0133
1100	0.0005 ± 0.0081, t(233) = -0.14972, p =0.8811; ω^2 = 0.0093
1200	-0.0111 ± 0.0063, t(233) = 4.3941, p <0.0001; ω^2 = 0.0099
1300	-0.0048 ± 0.0057, t(233) = 2.0878, p =0.0379; ω^2 = 0.0051
1400	-0.0146 ± 0.007, t(233) = 5.1816, p <0.0001; ω^2 = 0.0011
1500	-0.0259 ± 0.0071, t(233) = 9.0215, p <0.0001; ω^2 = -0.0062
1600	-0.0367 ± 0.0077, t(233) = 11.8365, p <0.0001; ω^2 = -0.0103
1700	-0.0302 ± 0.007, t(233) = 10.7248, p <0.0001; ω^2 = -0.0225
1800	-0.0325 ± 0.0068, t(233) = 11.9615, p <0.0001; ω^2 = 0.0216
1900	-0.0206 ± 0.006, t(233) = 8.4922, p <0.0001; ω^2 = 0.0336
2000	-0.0418 ± 0.0097, t(233) = 10.6327, p <0.0001; ω^2 = 0.0104
2100	0.002 ± 0.0072, t(233) = -0.69613, p =0.487; ω^2 = 0.0109

Table S4: Numerical values of maxima of burst rates.

frequency	measure	load 0	load 1	load 2	load 3
**alpha (8-12 Hz)**	mean	0.0999	0.0872	0.0843	0.1011
	+/- SEM	0.0046	0.0041	0.0042	0.0042
	at ms	698	1232	1256	1246
**beta (8-12 Hz)**	mean	0.088	0.0746	0.0776	0.0871
	+/- SEM	0.0036	0.0035	0.0035	0.0034
	at ms	705	1246	1241	1262
**low gamma (8-12 Hz)**	mean	0.0268	0.1044	0.0815	0.0771
	+/- SEM	0.0022	0.0015	0.0012	0.0013
	at ms	980	619	651	648
**high gamma (8-12 Hz)**	mean	0.0614	0.088	0.0819	0.0715
	+/- SEM	0.0025	0.002	0.0022	0.0021
	at ms	352	253	526	468

Table S5: Statistics of burst rates. Values of the 1-way ANOVA for load. Each row reports the extreme values of the significant phases indicated in Fig. 4A and Fig. S5.

**statistical values, extreme values**
**alpha (8-12 Hz)**
F(2,744) >= 3.052, p <= 0.0479, omega^2 range = [0.0055 0.1513]
**beta (13-19 Hz)**
F(2,744) >= 3.0136, p <= 0.0497, omega^2 range = [0.0054 0.1497]
**low gamma (33-48 Hz)**
F(2,744) >= 3.3732, p <= 0.0348, omega^2 range = [0.0063 0.2191]
**high gamma (83-98 Hz)**
F(2,744) >= 3.0116, p <= 0.0498, omega^2 range = [0.0054 0.1743]

Table S6: Intervals with significant difference for burst rate of original data vs. burst rate of surrogate data. Burst rate was considered significantly different if a t-test on burst rates indicated a significant difference for a consecutive period of two cycles of the respective bands center frequency, at an alpha level of 5 %.

frequency band	significant interval [start end] (ms) Load 1	significant interval [start end] (ms) Load 2	significant interval [start end] (ms) Load 3
**alpha (8-12 Hz)**			
	[2 821]	[2 862]	[2 564]
	[1052 1200]	[1094 1226]	[887 1242]
	[1422 1600]	[1460 1600]	[1501 1600]
**beta (13-19 Hz)**			
	[2 564]	[2 611]	[2 696]
	[1072 1263]	[1064 1330]	[1051 1379]
	[1420 1674]	[1512 1674]	[1619 1674]
**low gamma (33-48 Hz)**			
	[2 193]	[2 294]	[2 374]
	[256 892]	[436 892]	[447 889]
	[1044 1341]	[1055 1415]	[1038 1422]
	[1711 1750]	[1739 1750]	[1699 1750]
**high gamma (83-98 Hz)**			
	[6 124]	[3 131]	[2 70]
	[151 352]	[178 1021]	[99 161]
	[383 1014]	[1059 1778]	[199 736]
	[1060 1778]		[800 868]
			[895 1011]
			[1060 1778]
